# Supplementary material for: SARS-CoV-2 infection and antibody seroprevalence in routine surveillance patients, healthcare workers and general population in Kita region, Mali: an observational study 2020–2021
Source: BMJ Open. 2022 Jun 16;12(6):e060367. doi: 10.1136/bmjopen-2021-060367 (PMC9207578; doi:10.1136/bmjopen-2021-060367)
Supplement: Author's manuscript [file bmjopen-2021-060367.draft_revisions.pdf]

BMJ Open is committed to open peer review. As part of this commitment we make the peer review history of every article we publish publicly available.

When an article is published we post the peer reviewers' comments and the authors' responses online. We also post the versions of the paper that were used during peer review. These are the versions that the peer review comments apply to.

The versions of the paper that follow are the versions that were submitted during the peer review process. They are not the versions of record or the final published versions. They should not be cited or distributed as the published version of this manuscript.

BMJ Open is an open access journal and the full, final, typeset and author-corrected version of record of the manuscript is available on our site with no access controls, subscription charges or pay-per-view fees (http://bmjopen.bmj.com).

If you have any questions on BMJ Open's open peer review process please email info.bmjopen@bmj.com

BMJ Open

# SARS-CoV-2 infection and antibody seroprevalence in routine surveillance patients, health care workers and general population in Kita region, Mali



| | |
|---|---|
| Journal: | *BMJ Open* |
| Manuscript ID | bmjopen-2021-060367 |
| Article Type: | Original research |
| Date Submitted by the Author: | 19-Dec-2021 |



| | |
|---|---|
| Complete List of Authors: | Alber, Dagmar; Great Ormond Street Institute of Child Health, University College London<br>Haidara, Fadima; Center for Vaccine Development–Mali<br>Luoma, Juho; Tampere University, Center for Child, Adolescent and Maternal Health Research<br>Adubra, Laura; Tampere University, Center for Child, Adolescent and Maternal Health Research, Faculty of Medicine and Health Technology<br>Ashorn, Per; University of Tampere, Center for Child, Adolescent and Maternal Health Research, Faculty of Medicine and Health Technology; Tampere University Hospital, Department of Pediatrics<br>Ashorn, Ulla; University of Tampere, Center for Child, Adolescent and Maternal Health Research, Faculty of Medicine and Health Technology<br>Badji, Henry; Center for Vaccine Development–Mali<br>Cloutman-Green, Elaine; Great Ormond Street Hospital, Microbiology<br>Diallo, Fatoumata ; Center for Vaccine Development–Mali<br>Ihamuotila, Rikhard ; Tampere University, Center for Child, Adolescent and Maternal Health Research, Faculty of Medicine and Health Technology<br>Klein, Nigel; Institute Of Child Health,<br>Martell, Owen ; Tro Da Ltd<br>Onwuchekwa, Uma; Center for Vaccine Development–Mali<br>Samaké, Oumar; Center for Vaccine Development–Mali<br>Sow, Samba O.; Center for Vaccine Development–Mali<br>Traore, Awa; Center for Vaccine Development–Mali<br>Wilson, Kevin; Tampere University, Centre for Child, Adolescent and Maternal Health Research, Faculty of Medicine and Health Technology<br>Ducker, Camilla; Tro Da Ltd<br>Fan, Yuemei; Tampere University, Center for Child, Adolescent and Maternal Health Research |



| | |
|---|---|
| Keywords: | COVID-19, EPIDEMIOLOGY, Respiratory infections < THORACIC MEDICINE |
| | |


SCHOLARONE™
Manuscripts

*I, the Submitting Author has the right to grant and does grant on behalf of all authors of the Work (as defined in the below author licence), an exclusive licence and/or a non-exclusive licence for contributions from authors who are: i) UK Crown employees; ii) where BMJ has agreed a CC-BY licence shall apply, and/or iii) in accordance with the terms applicable for US Federal Government officers or employees acting as part of their official duties; on a worldwide, perpetual, irrevocable, royalty-free basis to BMJ Publishing Group Ltd ("BMJ") its licensees and where the relevant Journal is co-owned by BMJ to the co-owners of the Journal, to publish the Work in this journal and any other BMJ products and to exploit all rights, as set out in our licence.*

*The Submitting Author accepts and understands that any supply made under these terms is made by BMJ to the Submitting Author unless you are acting as an employee on behalf of your employer or a postgraduate student of an affiliated institution which is paying any applicable article publishing charge ("APC") for Open Access articles. Where the Submitting Author wishes to make the Work available on an Open Access basis (and intends to pay the relevant APC), the terms of reuse of such Open Access shall be governed by a Creative Commons licence – details of these licences and which Creative Commons licence will apply to this Work are set out in our licence referred to above.*

*Other than as permitted in any relevant BMJ Author's Self Archiving Policies, I confirm this Work has not been accepted for publication elsewhere, is not being considered for publication elsewhere and does not duplicate material already published. I confirm all authors consent to publication of this Work and authorise the granting of this licence.*

For peer review only

# SARS-CoV-2 infection and antibody seroprevalence in routine surveillance patients, health care workers and general population in Kita region, Mali

Dagmar Alber,[1]* Fadima Cheick Haidara,[2]* Juho Luoma,[3] Laura Adubra,[3] Per Ashorn,[3,4] Ulla Ashorn,[3] Henry Badji,[2] Elaine Cloutman-Green,[1] Fatoumata Diallo,[2] Rikhard Ihamuotila,[3] Nigel Klein,[1] Owen Martell,[5] Uma Omwuchekwa,[2] Oumar Samaké,[2] Samba Sow,[2] Awa Traore,[2] Kevin Wilson,[3] Camilla Ducker,[5#] Yue-Mei Fan[3#]

[1]Great Ormond Street Institute of Child Health, University College London, UK
[2]Center for Vaccine Development–Mali, Bamako, Mali
[3]Center for Child, Adolescent and Maternal Health Research, Faculty of Medicine and Health Technology, Tampere University, Tampere, Finland
[4]Department of Paediatrics, Tampere University Hospital, Tampere, Finland
[5]Tro Da Ltd, UK

*Co-First Authors, contributed equally
[#]Co-Last Authors, contributed equally

**Address for correspondence:**
Yue-Mei Fan, PhD
Centre for Child, Adolescent and Maternal Health Research, Faculty of Medicine and Health Technology, Tampere University, Tampere, Finland
Kauppi Campus, Arvo Ylpön katu 34
33520 Tampere, Finland
Email: yuemei.fan@tuni.fi (ORCID 0000-0002-3241-6225)

For peer review only

# Abstract

## *Objective*

To estimate the degree of severe acute respiratory syndrome coronavirus-2 (SARS-CoV-2) transmission among health care workers (HCWs) and general population in a West African setting.

## *Design*

Routine surveillance in twelve health facilities, HCWs serosurvey in five health facilities and community serosurvey in sixteen villages in or near Kita town, Mali.

## *Setting*

Kita region, western Mali; local health centres around the central (regional) referral health centre.

## *Participants*

Patients in routine surveillance, HCWs in local health centres and community members of all ages in populations associated with study health centres.

## *Main outcome measures*

Seropositivity of ELISA test detecting SARS-CoV-2-specific total antibodies and real time RT-PCR confirmed SARS-CoV-2 infection.

## *Results*

From 2392 routine surveillance at health facilities, 68 samples (2.8%, 95% CI: 2.2% to 3.6%) tested positive for SARS-CoV-2 by RT-PCR. The monthly positivity rate was 0% in June-August 2020 and gradually increased to 6% by December 2020 and 6.2% by January 2021, then declined to 5.5%, 3.3%, 3.6% and 0.8% in February, March, April and May 2021, respectively. From 397 serum samples collected from 113 HCWs, 175 (44.1%, 95% CI: 39.1% to 49.1%) were positive for SARS-CoV-2 antibodies. The monthly seroprevalence was around 10% from September to November 2020 and increased to over 40% from December 2020 to May 2021. For community serosurvey in December 2020, overall seroprevalence of SARS-CoV-2 antibodies was 27.7%. The highest age-stratified seroprevalence was observed in participants aged 60-69 years (45.5%, 95% CI: 32.3% to 58.6%). The lowest was in children aged 0-9 years (14.0%, 95% CI: 7.4% to 20.6%). Attendance at mass gatherings within three months of sample collection was associated with higher seropositivity (RR=1.48, 95% CI: 1.09 to 2.02).

## *Conclusions*

Community spread of SARS-CoV-2 infection started in Kita in October-November 2020, peaked in January 2021 and continued in May 2021. The observation is contrary to the widely expressed view, based on limited data, that Covid-19 infection rates were lower in 2020-2021 in West Africa than in other settings.




For peer review only - http://bmjopen.bmj.com/site/about/guidelines.xhtml

For peer review only



For peer review only - http://bmjopen.bmj.com/site/about/guidelines.xhtml

## Strengths and limitations of this study

- Our study was strengthened by the data available on participants from health facility clients, HCWs and the general population.
- We investigated both active infection and seroprevalence for HCWs and the general population during the same time period.
- First study of its kind in West Africa to provide routine surveillance data capturing.
- Our study was performed in one region and therefore it only reflects the Covid-19 situation of the Kita region.
- Extending active infection RT-PCR testing to non-symptomatic cases, both at the health facility and in the general population, would have provided more robust insights on infection rates as well as the level of asymptomatic cases.

## Funding

The study was funded by Bill & Melinda Gates Foundation, grant number INV-017141. The funders had no role in study design, data collection and analysis, decision to publish, or preparation of the manuscript.

## Competing interests

All authors have completed the ICMJE uniform disclosure form at www.icmje.org/coi_disclosure.pdf and declare: PA had financial support from Bill & Melinda Gates Foundation for the submitted work; no financial relationships with any organisations that might have an interest in the submitted work in the previous three years; no other relationships or activities that could appear to have influenced the submitted work.



For peer review only - http://bmjopen.bmj.com/site/about/guidelines.xhtml

# Introduction

The global coronavirus disease 2019 (Covid-19) pandemic, caused by the severe acute respiratory syndrome coronavirus 2 (SARS-CoV-2), has had a devastating impact on the health of populations in all regions of the world, leading to over five million deaths and tens of millions of cases, also causing widespread and long-term social and economic damage. Despite predictions made during the so-called "first wave" (March-May 2020) that Covid-19 would spread widely and cause untold damage to communities in African countries, data during that period suggested that transmission, case, and death rates have been lower in African settings than in other regions and continents.[1]

Whereas the reported numbers of SARS-CoV-2 infection have been low in large parts of Africa, there is some evidence that this may be due to undertesting or underreporting. A recent Zambian post-mortem study suggested that many people who die have an undiagnosed SARS-CoV-2 infection.[2] Unfortunately, there have been very few community-based SARS-CoV-2 studies published so far from Africa and hence the population prevalence in the continent is still largely unknown.

Our study was planned to characterise the epidemiology of Covid-19 epidemic in Kita region in western Mali, an area with very low rates of reported infections. To this end, we monitored time-trends in the proportion of positive SARS-CoV-2 virus test results among patients presenting with suspected SARS-CoV-2 infection in primary health centres over a period of 10 months and complemented the data with seropositivity for SARS-CoV-2 antibodies among personnel working in the same facilities during the same time frame. In the middle of the follow-up period, we conducted a population based viral and serological survey, to establish a point-prevalence of acute infection and past exposure to it, among apparently healthy people of different ages.



For peer review only - http://bmjopen.bmj.com/site/about/guidelines.xhtml

# Methods

## Study design and participants

The present study was designed to support the Malian Ministry of Health and Social Affairs' strategy to strengthen epidemiological surveillance and enhance the national response in Mali. The study was conducted in Kita region, Western Mali. The area is served by one referral health centre in Kita centre, while most health areas have a community health centre, offering a range of basic medical services with fee charged.

Our study targeted three groups –patients at a health facility, HCWs and apparently healthy community residents. We conducted surveillance for patients presenting at 1 referral health centre and 11 community health centres, over a period of 12 months. We conducted a HCWs serosurvey in five of the same health facilities over a period of 9 months and community serosurvey at one time point in 16 villages. The HCWs samples were from nurses, doctors, office workers, laboratory personnel and other auxiliary staff. The community samples were selected on the basis of cluster sampling. We used enumeration areas as clusters and determined the number of households from each cluster based on its recorded population size. The actual households to be invited were selected by random sampling.

## Data and Specimen collection

For the patient surveillance study, the study nurses filled in a patient screening form, a suspected infection enrolment form on exposures and symptoms and consenting form, followed by a medical examination and a flocked nasopharyngeal swab (NPS, flexible minitip, Copan) or oropharyngeal swab (OPS, flexible minitip, Copan; nasal and throat flocked swab, Dewei Medical Equipment Co.,



For peer review only - http://bmjopen.bmj.com/site/about/guidelines.xhtml

Ltd) specimen collection from those with a suspected SARS-CoV-2 infection, eg. patients experiencing fever, cough, shortness of breath and seeking medical advice. For the HCWs sero-surveillance study, study nurses filled in a screening, consent and enrolment form, a suspected infection enrolment form on symptoms and exposure questionnaire. Four ml of venous blood sample (BD vacutainer, Cat. No. 369032) was collected monthly and an NPS or OPS was taken at enrolment and every 12 weeks thereafter. From each community survey participant, the nurse/data collector filled in a symptom and exposure questionnaire and collected a nasal swab specimen and a blood sample. A study nurse or a data collector filled out the symptom and exposure questionnaire using a REDCap electronic data capture application. The biological samples were collected by a doctor at the health centre and by a nurse in the community survey. The collected swab samples were transported at 2-8 °C in 3 ml PBS/VTM from Kita to Bamako lab for analysis. The blood samples were centrifuged, and serum was aliquoted into cryovials at the Kita lab and batched samples were transported in dry shipper to Bamako for analysis.

## Nucleic acid extraction and real time RT-PCR

RNA was extracted from sample swabs using a QIAamp Viral RNA Mini kit (Qiagen, Germany, Cat. No. 52906) according to the manufacturer's instructions. Samples were eluted in a final volume of 140 µL.

Real time reverse transcription polymerase chain reaction (rtRT-PCR) assays for SARS-CoV-2 were carried out at the CVD-Mali Influenza laboratory, a participant in the WHO External Quality Assessment for the Detection of Influenza and SARS-CoV2 viruses. Briefly, extracted RNA was amplified using the Superscript One-step RT-PCR kit (Invitrogen, Thermo Fischer Scientific, Cat No. 11732-088) targeting the RdRp gene (Corman VM, 2020) or the Quantabio qScript XLT One-Step



For peer review only - http://bmjopen.bmj.com/site/about/guidelines.xhtml

RT-qPCR ToughMix targeting the N-gene (N2 primers) (CDC assay, https://www.fda.gov/media/134922/download).

Reactions (25 µL) were set up as follows: 2x master mix, 0.6 µM forward primer, 0.8 µM of reverse primer, 0.1µM of each probe, distilled-deionised (dd) $H_2O$and 5 µL of template. Reactions were cycled on a 7500 Fast ABI system (Applied Biosystems™) with an initial denaturation step at 95°C for 3 min followed by 45 cycles at 95°C for 30 s and 55°C for 30 s for the Superscript One-step RT-PCR kit, and 95°C for 15 s and 58°C for 30 s for the Quantabio qScript XLT One-Step RT-qPCR ToughMix.

When using the Takara Prime Direct RT-PCR (Takara Bio, Cat. No. RR650B and RR65TW), samples collected either in PBS or VTM were heated at 95°C for 10 min and used directly in the RT-PCR reaction. The Takara Prime Direct RT-PCR reaction mix consisted of Prime Takara master mix, 0.25 µM primers, 0.13 µM probe, dd$H_2O$ and 5 µL of template. Cycling conditions were as follows: 90°C for 3 min, 60°C for 5 min, and 45 cycles of 95°C for 5 s and 60°C for 30 s.

All assays were validated using WHO RNA samples received as part of an external Quality Assessment Programme and a panel of known positive and negative samples from University College London, UK.

## Serological assays

A laboratory technician performed the Wantai anti-SARS-Cov-2 total antibody ELISA (Beijing Wantai Biological Pharmacy Ent, Beijing, China) following the manufacturer's instructions. Serum samples (100 µL) from each participant were tested as a singlet. Ten batches were performed for community survey samples, six batches for HCW samples and one for repeated samples.

Assay validation was carried out using the WHO international standard (NIBSC code: 20/136), the WHO International Reference Panel for anti-SARS-CoV-2 immunoglobulin (NIBSC code: 20/268),



For peer review only - http://bmjopen.bmj.com/site/about/guidelines.xhtml

and an anti-SARSCoV-2 Verification Panel for Serology Assays (NIBSC code: 20/B770). In addition, 100 pre-pandemic samples collected in 2012 were tested to assess the specificity of assay. All of these pre-pandemic serum samples were from adult women (mean age 26 years old, range 16-41). Following the manufacturer's instructions, samples giving an absorbance equal to or greater than the cut-off value (A/CO >=1) were considered positive. Samples with A/CO ratio between 0.9 and 1.1 were considered borderline and were retested.

## Statistical analysis

The sample size was originally calculated to estimate the seroprevalence of SARS-Cov-2 antibodies with an absolute precision (margin of error) of 2.5% and assuming a baseline prevalence of 5%. Accounting for clustering effects of households within villages and of individuals within households, we estimated to require a minimum sample size of 3000. Assuming an average village population of 1446 (based on available regional statistics), average household size of 14, and average number of under-5 children per household of 3, we decided to visit 215 households to enrol the study sample.

We compared proportions, means and standard deviations (SD) between groups by using Student's *t* test for continuous variables and Fisher's exact test for proportions. We calculated risk ratios (RR) and 95% confidence intervals (CI) with regression model. All statistical analyses were carried out using R versions 3.4.4 and 4.1.0.



For peer review only - http://bmjopen.bmj.com/site/about/guidelines.xhtml

## Patient and public involvement

Village chefs and health centre leaders were consulted before the study to ensure feasibility of the planned study procedures. Participants were not involved in the development of the research design, conduct of the research or preparation of the manuscript.

## Results

Between June 2020 and May 2021, a total of 13,104 people presenting at outpatient clinics or using preventive services at 12 health facilities in or near the town of Kita were screened for exposure or symptoms of SARS-CoV-2 infection. Of these, 2393 were deemed to have a suspected SARS-CoV-2 infection and 2392 agreed to provide nasopharyngeal or oropharyngeal samples for viral detection.

From all the tested samples from health centre patients, 68 (2.8%, 95% CI 2.2% - 3.6%) gave a positive test result for SARS-CoV-2 by RT-PCR. Prior to August 2020, no SARS-CoV-2 RNA could be detected, infection rates steadily rose from November 2020 onwards, peaking at 6.2% (95% CI 3.3% - 10.4%) in January 2021, and then gradually declined to 0.8% (95% CI 0.02% - 4.1%) in May 2021 (Figure 1). As shown in the figure, the increase in the proportion of positive samples followed a similar time trend in nationally reported numbers of SARS-CoV-2 infections and associated deaths.

Between September 2020 and May 2021, a total of 113 members of staff were approached in five health facilities and invited to participate in the HCWs SARS-CoV-2 serosurvey. All 113 invited staff members agreed to participate. Among them, 89 (78.8%) were nurses, 9 (7.9%) were doctors, 7



For peer review only - http://bmjopen.bmj.com/site/about/guidelines.xhtml

(6.2%) were administrative or laboratory technicians, and 8 (7.1%) were auxiliary staff including housekeepers, cleaners and security staff.

Over the 9-month follow-up period, the participants provided a total of 397 blood samples, with a range of 1-7 samples per participant. Of these samples, 175 (44.1%, 95% CI 39.1% - 49.1%) were positive for SARS-CoV-2 antibody by ELISA. By calendar month, the proportion of positive samples was approximately 10% until November 2020, increasing rapidly to 59.1% (95% CI 38.5% - 79.6%) in January 2021 and remained relatively constant thereafter (Figure 2). For different health professional groups, the proportion of SARS-CoV-2 antibody positive samples was highest among doctors (50.0%, 95% CI 29.9% - 70.1%, 13/26 samples), followed by 49.8% (95% CI 43.4% - 56.2%, 124/249) among nurses, 35.9% (95% CI 21.2% - 52.8%, 14/39) among administrative staff and laboratory technicians and 15.4% (95% CI 1.9% - 45.5%, 2/13) among auxiliary staff.

Of the 101 NPS or OPS samples collected from the health personnel, only one (1.0%) collected in November 2020 gave a positive test result for SARS-CoV-2 by RT-PCR.

In December 2020, a total of 229 randomly selected households were visited in villages in or near the Kita centre and invited to participate in a SARS-CoV-2 community survey. Heads of all the invited households provided a consent and a total of 905 participants were included in the survey. Of the participants, 564 (62.3%) were women, 654 (72.3%) were at least 15 years old, 102 (11.3%) had respiratory symptoms in the preceding 14 days, 496 (55.4%) had attended a mass gathering in the preceding 3 months, none reported a contact with a Covid-19 patient, and 21 (2.3%) had a fever.

Of the 905 community survey participants, 882 provided a blood sample for antibody analysis and 894 provided a nasal swab for viral detection. Among all the participants, the overall



For peer review only - http://bmjopen.bmj.com/site/about/guidelines.xhtml

seroprevalence was 27.7% (95% CI 24.7% – 30.8%). The seroprevalence was lowest (14.0%, 95% CI: 7.4% - 20.6%) among 0-9-year-old and highest (45.5%, 95% CI: 32.3% to 58.6%) among 60-69-year-old participants (Figure 3). Participants who had attended a mass gathering in the preceding 3 months had a higher seroprevalence than those who had not (relative risk 1.48, 95% CI: 1.09 - 2.02). Participant sex, measured fever, and recent history of fever or other respiratory symptoms were not associated with the presence of SARS-CoV-2 antibodies in the blood samples (Table 1).

A total of 21 (2.4%, 95% CI 1.5% – 3.6%) community survey participants had a positive RT-PCR result for SARS-CoV-2. The proportion of positive SARS-CoV-2 PCR tests was lowest (0.4%, 95% CI: 0% - 1.3%) among 10-19-year-old and highest (5.8%, 95% CI: 0.3% - 11.3%) among 50-59-year-old participants (Figure 3).

Of the 100 banked serum samples collected before the Covid-19 pandemic (in year 2012), one gave a borderline positive result and 99 gave a negative test result in the Wantai SARS-CoV-2 antibody test.

## Discussion

This study measured active and past Covid-19 infection in Malian HCWs and the general population through RT-PCR and seroprevalence testing. The results point towards significant underestimation of Mali’s Covid-19 infection rates, as measured by current testing mechanisms. The study also provides insights into Covid-19 transmission patterns and risk factors. Extremely high infection rates were demonstrated by the study’s serological testing. By December 2020 over a quarter (27.7%) of Kita’s general population, and more than a third (41.9%) of HCWs had been infected. By January 2021, this had increased to 60% of HCWs.

For peer review only

National routine surveillance morbidity data for the study period (June 2020 – May 2021), indicates that in June, transmission was at its first-wave peak, and that two later larger waves occurred, peaking in early December 2020 and April 2021.[3] Both the study's facility-based client/patient active infection investigation and HCWs seroprevalence findings indicate that Kita region's main transmission started later, in November 2020. This suggests that the region escaped the earlier wave, with Covid-19 imported sometime in late October/early November, perhaps around the celebrations for the Prophet Mohamed's birthday on 29$^{th}$ October. Both study groups indicate a peak in December/January, similar to that seen nationally. The clients' active infection rate then showed a gradual decline to May rather than a second wave in March that was seen nationally.[3] This could be explained by containment measures reducing the emergence of a second peak in the general population.

A comparison of this study's seroprevalence and active infection rates show that seroprevalence is much higher than active cases in both the study's general population and in HCWs. The fact that this high seroprevalence did not translate into a similar expression of morbidity and resultant death, neither in our study nor in the national surveillance system is an interesting area to investigate. This scenario has been documented quite widely in Africa.[4,5] This could be explained by a multitude of interlinking factors. High levels of asymptomatic cases, both for the general population and HCWs, have been documented in African as well as global studies.[6-11] As our study's active infection testing selected suspected cases of Covid-19 only, we could have missed a large proportion of asymptomatic cases. National surveillance would also have missed these cases as, firstly, they wouldn't present to a health centre and, secondly, even those that did would have been missed by diagnostic protocols.



For peer review only - http://bmjopen.bmj.com/site/about/guidelines.xhtml

Mali has gaps in demographic, disease morbidity, and death surveillance, including for Covid-19. A recent Zambian study showed that nearly 20% of people who had died had a positive SARS-CoV-2 PCR test in a postmortem NP swab sample. These people had not been tested before death, even though some of them had presented with Covid-19 symptoms.[2] A similar situation is likely present in Mali where diagnostic capacity is limited, at 7191 tests per 1 million people (compared to that of South Africa, 113 756) (Salyer, ym., 2021), and where Covid-19 stigma exists.[12,13]

This study showed that 1.5 times more HCWs in Kita had been infected with SARS-CoV-2 by December 2020 compared to the general population. In fact, half of patient facing cadres such as doctors and nurses had been exposed to the virus and this was higher than laboratory, administrative (both 36%) and auxiliary staff (15.4%). Measures of increased risk of infection in HCWs vs the general population have been demonstrated in many global studies, with adequate PPE provision and use being key to minimizing this risk.[4,6,11,14] In resource-constrained settings such as Kita, key tools for protecting HCWs, along with early disease detection, are often limited or absent in Africa.[15,16] Measures to improve protection for doctors and nurses, especially considering low levels of health staff availability in the country – in 2017 Mali had only six qualified providers per 10,000 inhabitants – would be pertinent.[17]

In the general population, risk factors included increased age and attendance at mass gatherings. Seroprevalence was lowest in the very young, and highest in 60-69-year-olds but again lower in the 70+ year olds. Mass gatherings are likely sources of infection given Covid-19's transmission dynamics and the difficulty of ensuring appropriate prevention control measures (mask wearing, social distancing, hand washing) at such events. Interestingly, neither fever (current or recent) nor other respiratory symptoms were seen to be significant risk factors, which again infers a high asymptomatic population.

For peer review only



For peer review only - http://bmjopen.bmj.com/site/about/guidelines.xhtml

The study's principal strength is that we included health facility clients, HCWs and the general population. We also investigated both active infection and seroprevalence for HCWs and the general population during the same time period. Study limitations include restriction to one region and therefore this study only reflects the Covid-19 situation of the Kita region. Extending active infection RT-PCR testing to non-symptomatic cases, both at the health facility and in the general population, would have provided more robust insights on infection rates as well as the level of asymptomatic cases. Widening the study to investigate exposure/transmission factors for study participants, such as PPE provision and use, social distancing, Covid-19 interventions, and behaviour change would have allowed the study to explore risk factors to better inform policy. When interpreting the RT-PCR results, possible sources of bias could have occurred within the sample collection regarding sample types (oropharyngeal/nasopharyngeal swabs), sample quality, transportation and temperature, and also in the use of 3 different RT-PCR assays. The latter is unlikely to have affected the results as all RT-PCR assays were validated in-house and by others.[18] Previous reports have suggested that some SARS-CoV-2 ELISA kits lack specificity when used on serum samples originating from Africa and in particular exhibit pre-existing cross-reactivity to SARS-CoV-2.[19,20] Our assay showed a 99% specificity when pre-pandemic samples were analysed and previous work has shown the Wantai assay to be highly sensitive (93%) and specific (100%).[21]

The main finding of this study is that SARS-CoV-2 in rural Mali is much more widespread than assumed by national testing data and particularly in the older population and frontline health workers. Institutional preparedness as well as public risk assessment and personal protection capacity should be augmented, and behaviours in high-risk activities such as mass gatherings improved, if cessation of such events is not possible. Improving the understanding of Covid-19 in Mali through surveillance will enable the country to reduce the economic and social impacts of the disease. Further studies which provide accurate estimates of SARS-CoV-2 infection rates and



For peer review only - http://bmjopen.bmj.com/site/about/guidelines.xhtml

seroconversion in the population would enable the development of a national transmission model, mapping of transmission hotspots, identification of national/regional risk factors, and the prioritization of equitable and effective responses. Such studies could also direct policy determinations in terms of disease prioritization, ensuring that resources reallocated for Covid-19 control measures are used appropriately.

For peer review only



For peer review only - http://bmjopen.bmj.com/site/about/guidelines.xhtml

## Contributors

DA and FCH contributed equally and share first authorship. CD and YMF contributed equally and share last authorship. JL performed statistical analysis. PA, UA, NK, SS, KW, CD and YMF initiated the project and preliminary design. DA, FCH, LA, HB, ECG, FD, RI, OM, UM, OS, AT developed the design and methods. DA, FCH, CD and YMF wrote the first draft of the manuscript. All authors contributed to the design of the study and interpretation of its results, and revised and approved the manuscript. PA and YMF are the guarantors and accept full responsibility for the work and the conduct of the study, had access to the data, and controlled the decision to publish. The corresponding author attests that all listed authors meet authorship criteria and that no others meeting the criteria have been omitted.

## Data sharing

Deidentified participant data might be available on reasonable request to the corresponding author.

## Ethics

The study protocol was approved by the Faculté de Médecine, Pharmacie et d'Odonto-stomatologie (FMPOS) institutional review board in Mali and only participants who provided informed consent were enrolled in the study. All consents were requested verbally and recorded in the electronic data capture system. The minors' caregivers gave consent for their children to participate in the study.

For peer review only



For peer review only - http://bmjopen.bmj.com/site/about/guidelines.xhtml

## References

1 COVID-19 Map. Johns Hopkins Coronavirus Resource Center. https://coronavirus.jhu.edu/map.html (accessed 10 Nov 2021).

2 Mwananyanda L, Gill CJ, MacLeod W, *et al.* Covid-19 deaths in Africa: prospective systematic postmortem surveillance study. *BMJ* 2021;:n334. doi:10.1136/bmj.n334

3 Mali: WHO Coronavirus Disease (COVID-19) Dashboard With Vaccination Data. https://covid19.who.int (accessed 10 Nov 2021).

4 Chibwana MG, Jere KC, Kamn'gona R, *et al.* High SARS-CoV-2 seroprevalence in health care workers but relatively low numbers of deaths in urban Malawi. *medRxiv* 2020;:2020.07.30.20164970. doi:10.1101/2020.07.30.20164970

5 Uyoga S, Adetifa IMO, Karanja HK, *et al.* Seroprevalence of anti–SARS-CoV-2 IgG antibodies in Kenyan blood donors. *Science* 2021;**371**:79–82. doi:10.1126/science.abe1916

6 Abatan B, Agboghoroma O, Akemoke F, *et al.* Intense and Mild First Epidemic Wave of Coronavirus Disease, The Gambia. *Emerg Infect Dis* 2021;**27**:2064–72. doi:10.3201/eid2708.204954

7 Gelanew T, Seyoum B, Mulu A, *et al.* High Seroprevalence of Anti-SARS-CoV-2 Antibodies Among Ethiopian Healthcare Workers. *Res Sq* 2021;:rs.3.rs-676935. doi:10.21203/rs.3.rs-676935/v1

8 Paleker M, Tembo YA, Davies M-A, *et al.* Asymptomatic COVID-19 in South Africa – implications for the control of transmission. *Public Health Action* 2021;**11**:58–60. doi:10.5588/pha.20.0069

9 Fai KN, Corine TM, Bebell LM, *et al.* Serologic response to SARS-CoV-2 in an African population. *Scientific African* 2021;**12**:e00802. doi:10.1016/j.sciaf.2021.e00802

10 Oran DP, Topol EJ. Prevalence of Asymptomatic SARS-CoV-2 Infection. *Ann Intern Med* 2020;**173**:362–7. doi:10.7326/M20-3012

11 Olayanju O, Bamidele O, Edem F, *et al.* SARS-CoV-2 Seropositivity in Asymptomatic Frontline Health Workers in Ibadan, Nigeria. *The American Journal of Tropical Medicine and Hygiene* 2021;**104**:91–4. doi:10.4269/ajtmh.20-1235

12 Salyer SJ, Maeda J, Sembuche S, *et al.* The first and second waves of the COVID-19 pandemic in Africa: a cross-sectional study. *The Lancet* 2021;**397**:1265–75. doi:10.1016/S0140-6736(21)00632-2

13 Seidu A-A, Hagan JE, Ameyaw EK, *et al.* The role of testing in the fight against COVID-19: Current happenings in Africa and the way forward. *International Journal of Infectious Diseases* 2020;**98**:237–40. doi:10.1016/j.ijid.2020.06.089



For peer review only - http://bmjopen.bmj.com/site/about/guidelines.xhtml

14 Pollán M, Pérez-Gómez B, Pastor-Barriuso R, *et al.* Prevalence of SARS-CoV-2 in Spain (ENE-COVID): a nationwide, population-based seroepidemiological study. *The Lancet* 2020;**396**:535–44. doi:10.1016/S0140-6736(20)31483-5

15 Galanis P, Vraka I, Fragkou D, *et al.* Seroprevalence of SARS-CoV-2 antibodies and associated factors in healthcare workers: a systematic review and meta-analysis. *J Hosp Infect* 2021;**108**:120–34. doi:10.1016/j.jhin.2020.11.008

16 Quadri NS, Sultan A, Ali SI, *et al.* COVID-19 in Africa: Survey Analysis of Impact on Health-Care Workers. *Am J Trop Med Hyg* 2021;**104**:2169–75. doi:10.4269/ajtmh.20-1478

17 M'Bo M, Tessougue J, International I, *et al.* DECENTRALIZING MALI'S HEALTH WORKFORCE INFORMATION SYSTEM. ;:4.

18 Brown JR, O'Sullivan DM, Shah D, *et al.* Comparison of SARS-CoV-2 N gene real-time RT-PCR targets and commercially available mastermixes. *J Virol Methods* 2021;**295**:114215. doi:10.1016/j.jviromet.2021.114215

19 Emmerich P, Murawski C, Ehmen C, *et al.* Limited specificity of commercially available SARS-CoV-2 IgG ELISAs in serum samples of African origin. *Trop Med Int Health* 2021;:10.1111/tmi.13569. doi:10.1111/tmi.13569

20 Tso FY, Lidenge SJ, Peña PB, *et al.* High prevalence of pre-existing serological cross-reactivity against severe acute respiratory syndrome coronavirus-2 (SARS-CoV-2) in sub-Saharan Africa. *Int J Infect Dis* 2021;**102**:577–83. doi:10.1016/j.ijid.2020.10.104

21 Lassaunière R, Frische A, Harboe ZB, *et al.* Evaluation of nine commercial SARS-CoV-2 immunoassays. 2020. doi:10.1101/2020.04.09.20056325


For peer review only



For peer review only - http://bmjopen.bmj.com/site/about/guidelines.xhtml

**Table 1. Seroprevalence of SARS-CoV-2-specific total antibody by background characteristics**

| | Sample size, n | Seropositive participants, n | Seroprevalence, % | OR (95% CI) |
|---|---|---|---|---|
| Participants, *n* | 875 | 242 | 27.7% | |
| Sex | | | | |
| Male | 330 | 98 | 29.7% | Ref |
| Female | 543 | 142 | 26.2% | 0.84 (0.62-1.14) |
| Fever during past 14 days | | | | |
| No fever | 832 | 229 | 27.5% | Ref |
| Fever | 41 | 11 | 26.8% | 0.97 (0.48-1.96) |
| Fever (body temperature over 38 °C) | | | | |
| No fever | 852 | 238 | 27.9% | Ref |
| Fever | 21 | 2 | 9.5% | 0.27 (0.06-1.17) |
| Respiratory symptoms in the past 14 days | | | | |
| No symptoms | 773 | 216 | 27.9% | Ref |
| Any symptoms | 100 | 24 | 24.0% | 0.81 (0.50-1.32) |
| Mass gatherings in the past 3 months | | | | |
| No attendance | 370 | 86 | 23.0% | Ref |
| Attendance | 487 | 151 | 31.0% | 1.48 (1.09-2.02) |

For peer review only

**Legends of Figures:**

**Figure 1.** Percentage of SARS-CoV-2 positive RT-PCR test by month in routine surveillance patients from health facilities. Grey area and values in parentheses indicate 95% confidence interval from the proportion of SARS-CoV-2 RNA containing samples. Yellow lines indicate nationally reported numbers of SARS-CoV-2 infections ((WHO https://covid19.who.int/region/afro/country/ml). Values above the x-axis indicate number of study samples in each month. PS = pharyngeal swab, SARS-CoV-2 = Severe acute respiratory syndrome coronavirus-2, RNA = ribonucleic acid.

**Figure 2.** Seroprevalence of SARS-CoV-2-specific total antibody by month in health care worker serosurvey. Grey area and values in parentheses indicate 95% confidence interval from the proportion of SARS-CoV-2-specific antibody containing samples. Values above the x-axis indicate number of study samples in each month. SARS-CoV-2 = Severe acute respiratory syndrome coronavirus-2.

**Figure 3.** Seroprevalence of SARS-CoV-2-specific total antibody and percentage of SARS-CoV-2 positive RT-PCR test by 10-year age interval group in community participants. Red bars indicate proportion (95% CI) of serum samples containing SARS-CoV-2-specific antibodies (primary y-axis), blue bars indicate proportion (95% CI) of PS samples containing SARS-CoV-2 RNA (secondary y axis). PS = pharyngeal swab, SARS-CoV-2 = Severe acute respiratory syndrome coronavirus-2, RNA = ribonucleic acid.



For peer review only - http://bmjopen.bmj.com/site/about/guidelines.xhtml

**Figure 1.**

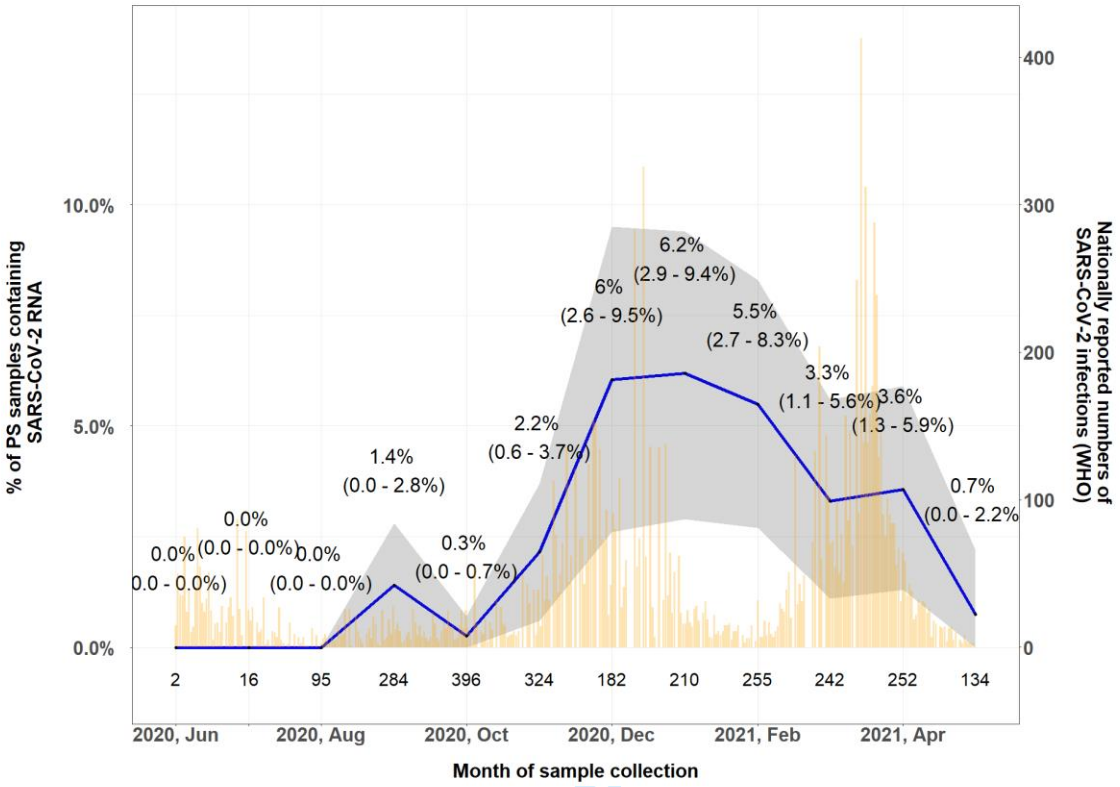


For peer review only

**Figure 2.**

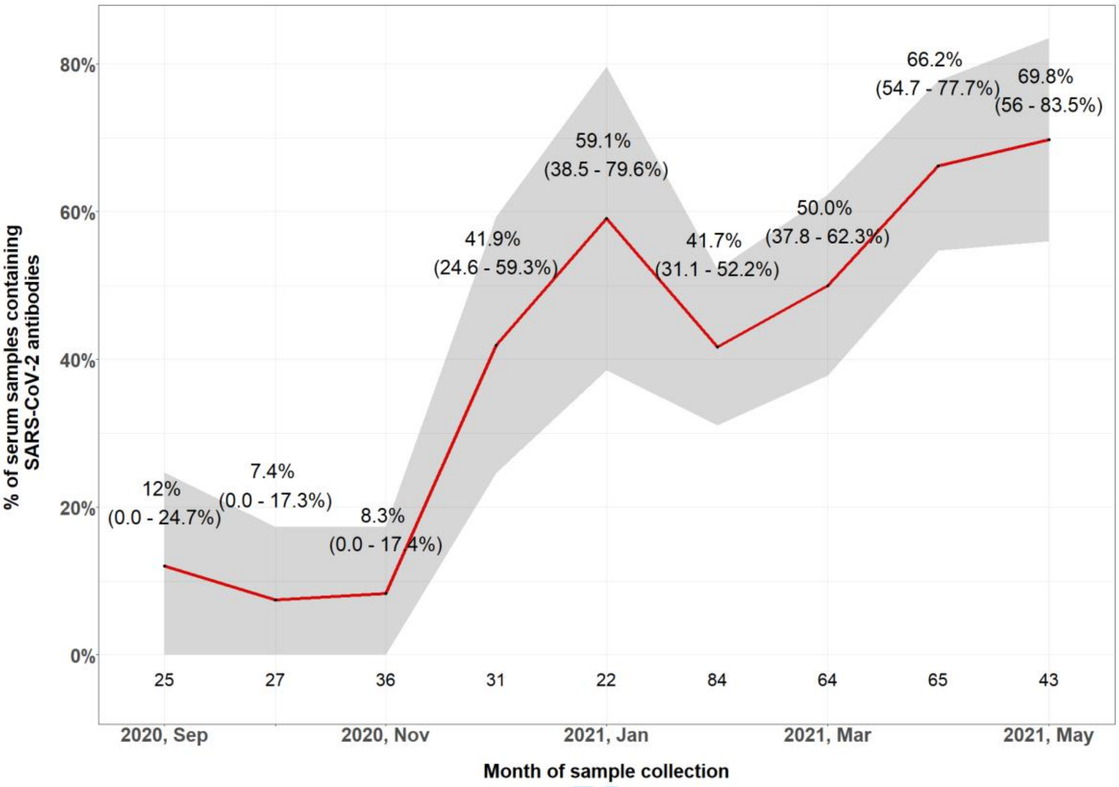
80%
60%
40%
20%
0%
% of serum samples containing SARS-CoV-2 antibodies
12%
(0.0 - 24.7%)
7.4%
(0.0 - 17.3%)
8.3%
(0.0 - 17.4%)
41.9%
(24.6 - 59.3%)
59.1%
(38.5 - 79.6%)
41.7%
(31.1 - 52.2%)
50.0%
(37.8 - 62.3%)
66.2%
(54.7 - 77.7%)
69.8%
(56 - 83.5%)
25
27
36
31
22
84
64
65
43
2020, Sep
2020, Nov
2021, Jan
2021, Mar
2021, May
Month of sample collection

**Figure 3.**

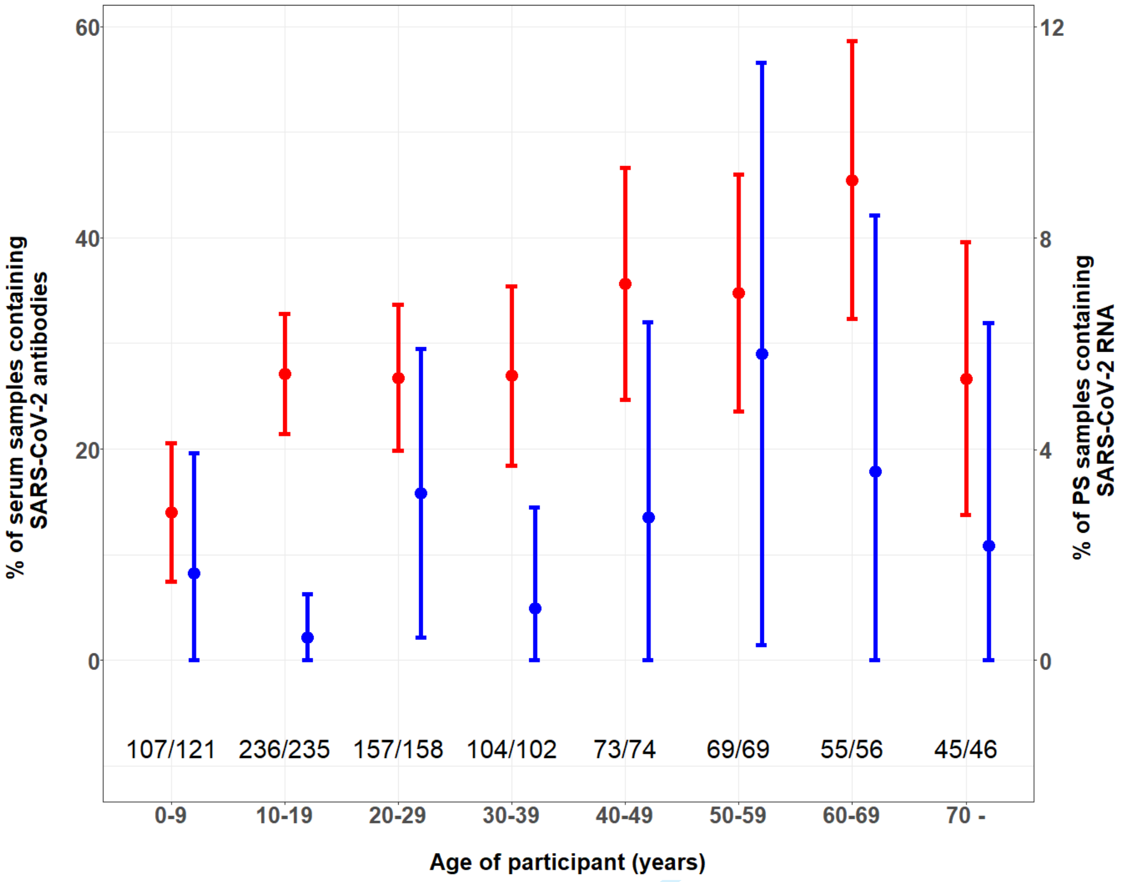
60
40
20
0
12
8
4
0
% of serum samples containing SARS-CoV-2 antibodies
% of PS samples containing SARS-CoV-2 RNA
107/121
236/235
157/158
104/102
73/74
69/69
55/56
45/46
0-9
10-19
20-29
30-39
40-49
50-59
60-69
70 -
Age of participant (years)

STROBE Statement—checklist of items that should be included in reports of observational studies

| | Item No | Recommendation | Page No |
|---|---|---|---|
| **Title and abstract** | 1 | (*a*) Indicate the study's design with a commonly used term in the title or the abstract | 2 |
| | | (*b*) Provide in the abstract an informative and balanced summary of what was done and what was found | 2 |
| **Introduction** | | | |
| Background/rationale | 2 | Explain the scientific background and rationale for the investigation being reported | 4 |
| Objectives | 3 | State specific objectives, including any prespecified hypotheses | 4 |
| **Methods** | | | |
| Study design | 4 | Present key elements of study design early in the paper | 5 |
| Setting | 5 | Describe the setting, locations, and relevant dates, including periods of recruitment, exposure, follow-up, and data collection | 5 |
| Participants | 6 | (*a*) *Cohort study*—Give the eligibility criteria, and the sources and methods of selection of participants. Describe methods of follow-up<br>*Case-control study*—Give the eligibility criteria, and the sources and methods of case ascertainment and control selection. Give the rationale for the choice of cases and controls<br>*Cross-sectional study*—Give the eligibility criteria, and the sources and methods of selection of participants | 5 |
| | | (*b*) *Cohort study*—For matched studies, give matching criteria and number of exposed and unexposed<br>*Case-control study*—For matched studies, give matching criteria and the number of controls per case | |
| Variables | 7 | Clearly define all outcomes, exposures, predictors, potential confounders, and effect modifiers. Give diagnostic criteria, if applicable | 5-8 |
| Data sources/ measurement | 8* | For each variable of interest, give sources of data and details of methods of assessment (measurement). Describe comparability of assessment methods if there is more than one group | *5-8* |
| Bias | 9 | Describe any efforts to address potential sources of bias | 5-8 |
| Study size | 10 | Explain how the study size was arrived at | 8 |
| Quantitative variables | 11 | Explain how quantitative variables were handled in the analyses. If applicable, describe which groupings were chosen and why | 8 |
| Statistical methods | 12 | (*a*) Describe all statistical methods, including those used to control for confounding | 8 |
| | | (*b*) Describe any methods used to examine subgroups and interactions | |
| | | (*c*) Explain how missing data were addressed | |
| | | (*d*) *Cohort study*—If applicable, explain how loss to follow-up was addressed<br>*Case-control study*—If applicable, explain how matching of cases and controls was addressed<br>*Cross-sectional study*—If applicable, describe analytical methods taking account of sampling strategy | |
| | | (*e*) Describe any sensitivity analyses | |

Continued on next page



For peer review only - http://bmjopen.bmj.com/site/about/guidelines.xhtml

**Results**

| | | | |
|---|---|---|---|
| Participants | 13* | (a) Report numbers of individuals at each stage of study—eg numbers potentially eligible, examined for eligibility, confirmed eligible, included in the study, completing follow-up, and analysed | 9-10 |
| | | (b) Give reasons for non-participation at each stage | |
| | | (c) Consider use of a flow diagram | |
| Descriptive data | 14* | (a) Give characteristics of study participants (eg demographic, clinical, social) and information on exposures and potential confounders | 9-10 |
| | | (b) Indicate number of participants with missing data for each variable of interest | |
| | | (c) *Cohort study*—Summarise follow-up time (eg, average and total amount) | |
| Outcome data | 15* | *Cohort study*—Report numbers of outcome events or summary measures over time | *9-10* |
| | | *Case-control study*—Report numbers in each exposure category, or summary measures of exposure | |
| | | *Cross-sectional study*—Report numbers of outcome events or summary measures | |
| Main results | 16 | (*a*) Give unadjusted estimates and, if applicable, confounder-adjusted estimates and their precision (eg, 95% confidence interval). Make clear which confounders were adjusted for and why they were included | |
| | | (*b*) Report category boundaries when continuous variables were categorized | |
| | | (*c*) If relevant, consider translating estimates of relative risk into absolute risk for a meaningful time period | |
| Other analyses | 17 | Report other analyses done—eg analyses of subgroups and interactions, and sensitivity analyses | |

**Discussion**

| | | | |
|---|---|---|---|
| Key results | 18 | Summarise key results with reference to study objectives | 11 |
| Limitations | 19 | Discuss limitations of the study, taking into account sources of potential bias or imprecision. Discuss both direction and magnitude of any potential bias | 13 |
| Interpretation | 20 | Give a cautious overall interpretation of results considering objectives, limitations, multiplicity of analyses, results from similar studies, and other relevant evidence | 11-13 |
| Generalisability | 21 | Discuss the generalisability (external validity) of the study results | 14 |

**Other information**

| | | | |
|---|---|---|---|
| Funding | 22 | Give the source of funding and the role of the funders for the present study and, if applicable, for the original study on which the present article is based | 15 |

*Give information separately for cases and controls in case-control studies and, if applicable, for exposed and unexposed groups in cohort and cross-sectional studies.

**Note:** An Explanation and Elaboration article discusses each checklist item and gives methodological background and published examples of transparent reporting. The STROBE checklist is best used in conjunction with this article (freely available on the Web sites of PLoS Medicine at http://www.plosmedicine.org/, Annals of Internal Medicine at http://www.annals.org/, and Epidemiology at http://www.epidem.com/). Information on the STROBE Initiative is available at www.strobe-statement.org.

For peer review only

BMJ Open

# SARS-CoV-2 infection and antibody seroprevalence in routine surveillance patients, health care workers and general population in Kita region, Mali: an observational study 2020 - 2021



| | |
|---|---|
| Journal: | *BMJ Open* |
| Manuscript ID | bmjopen-2021-060367.R1 |
| Article Type: | Original research |
| Date Submitted by the Author: | 18-Apr-2022 |



| | |
|---|---|
| Complete List of Authors: | Alber, Dagmar; Great Ormond Street Institute of Child Health, University College London<br>Haidara, Fadima; Center for Vaccine Development–Mali<br>Luoma, Juho; Tampere University, Center for Child, Adolescent and Maternal Health Research<br>Adubra, Laura; Tampere University, Center for Child, Adolescent and Maternal Health Research, Faculty of Medicine and Health Technology<br>Ashorn, Per; University of Tampere, Center for Child, Adolescent and Maternal Health Research, Faculty of Medicine and Health Technology; Tampere University Hospital, Department of Pediatrics<br>Ashorn, Ulla; University of Tampere, Center for Child, Adolescent and Maternal Health Research, Faculty of Medicine and Health Technology<br>Badji, Henry; Center for Vaccine Development–Mali<br>Cloutman-Green, Elaine; Great Ormond Street Hospital, Microbiology<br>Diallo, Fatoumata ; Center for Vaccine Development–Mali<br>Ihamuotila, Rikhard ; Tampere University, Center for Child, Adolescent and Maternal Health Research, Faculty of Medicine and Health Technology<br>Klein, Nigel; Institute Of Child Health,<br>Martell, Owen ; Tro Da Ltd<br>Onwuchekwa, Uma; Center for Vaccine Development–Mali<br>Samaké, Oumar; Center for Vaccine Development–Mali<br>Sow, Samba O.; Center for Vaccine Development–Mali<br>Traore, Awa; Center for Vaccine Development–Mali<br>Wilson, Kevin; Tampere University<br>Ducker, Camilla; Tro Da Ltd<br>Fan, Yuemei; Tampere University, Center for Child, Adolescent and Maternal Health Research |



| | |
|---|---|
| <b>Primary Subject Heading</b>: | Epidemiology |
| Secondary Subject Heading: | Infectious diseases, Global health |
| Keywords: | COVID-19, EPIDEMIOLOGY, Respiratory infections < THORACIC MEDICINE |
| | |

SCHOLARONE™
Manuscripts

*I, the Submitting Author has the right to grant and does grant on behalf of all authors of the Work (as defined in the below author licence), an exclusive licence and/or a non-exclusive licence for contributions from authors who are: i) UK Crown employees; ii) where BMJ has agreed a CC-BY licence shall apply, and/or iii) in accordance with the terms applicable for US Federal Government officers or employees acting as part of their official duties; on a worldwide, perpetual, irrevocable, royalty-free basis to BMJ Publishing Group Ltd ("BMJ") its licensees and where the relevant Journal is co-owned by BMJ to the co-owners of the Journal, to publish the Work in this journal and any other BMJ products and to exploit all rights, as set out in our licence.*

*The Submitting Author accepts and understands that any supply made under these terms is made by BMJ to the Submitting Author unless you are acting as an employee on behalf of your employer or a postgraduate student of an affiliated institution which is paying any applicable article publishing charge ("APC") for Open Access articles. Where the Submitting Author wishes to make the Work available on an Open Access basis (and intends to pay the relevant APC), the terms of reuse of such Open Access shall be governed by a Creative Commons licence – details of these licences and which Creative Commons licence will apply to this Work are set out in our licence referred to above.*

*Other than as permitted in any relevant BMJ Author's Self Archiving Policies, I confirm this Work has not been accepted for publication elsewhere, is not being considered for publication elsewhere and does not duplicate material already published. I confirm all authors consent to publication of this Work and authorise the granting of this licence.*

For peer review only

# SARS-CoV-2 infection and antibody seroprevalence in routine surveillance patients, health care workers and general population in Kita region, Mali: an observational study 2020 - 2021

Dagmar Alber,[1]* Fadima Cheick Haidara,[2]* Juho Luoma,[3] Laura Adubra,[3] Per Ashorn,[3,4] Ulla Ashorn,[3] Henry Badji,[2] Elaine Cloutman-Green,[1] Fatoumata Diallo,[2] Rikhard Ihamuotila,[3] Nigel Klein,[1] Owen Martell,[5] Uma Onwuchekwa,[2] Oumar Samaké,[2] Samba Sow,[2] Awa Traore,[2] Kevin Wilson,[3] Camilla Ducker,[5#] Yue-Mei Fan[3#]

[1]Great Ormond Street Institute of Child Health, University College London, UK
[2]Center for Vaccine Development–Mali, Bamako, Mali
[3]Center for Child, Adolescent and Maternal Health Research, Faculty of Medicine and Health Technology, Tampere University, Tampere, Finland
[4]Department of Paediatrics, Tampere University Hospital, Tampere, Finland
[5]Tro Da Ltd, UK

*Co-First Authors, contributed equally
[#]Co-Last Authors, contributed equally

**Address for correspondence:**
Yue-Mei Fan, PhD
Centre for Child, Adolescent and Maternal Health Research
Faculty of Medicine and Health Technology
Tampere University
Tampere, Finland
Kauppi Campus, Arvo Ylpön katu 34
33520 Tampere, Finland
Email: yuemei.fan@tuni.fi (ORCID 0000-0002-3241-6225)



For peer review only - http://bmjopen.bmj.com/site/about/guidelines.xhtml

# Abstract

## *Objective*

To estimate the degree of severe acute respiratory syndrome coronavirus-2 (SARS-CoV-2) transmission among health care workers (HCWs) and general population in Kita region of Mali.

## *Design*

Routine surveillance in twelve health facilities, HCWs serosurvey in five health facilities and community serosurvey in sixteen villages in or near Kita town, Mali.

## *Setting*

Kita region, western Mali; local health centres around the central (regional) referral health centre.

## *Participants*

Patients in routine surveillance, HCWs in local health centres and community members of all ages in populations associated with study health centres.

## *Main outcome measures*

Seropositivity of ELISA test detecting SARS-CoV-2-specific total antibodies and real time RT-PCR confirmed SARS-CoV-2 infection.

## *Results*

From 2392 routine surveillance samples, 68 (2.8%, 95% CI: 2.2% to 3.6%) tested positive for SARS-CoV-2 by RT-PCR. The monthly positivity rate was 0% in June-August 2020 and gradually increased to 6% by December 2020 and 6.2% by January 2021, then declined to 5.5%, 3.3%, 3.6% and 0.8% in February, March, April and May 2021, respectively. From 397 serum samples collected from 113 HCWs, 175 (44.1%, 95% CI: 39.1% to 49.1%) were positive for SARS-CoV-2 antibodies. The monthly seroprevalence was around 10% from September to November 2020 and increased to over 40% from December 2020 to May 2021. For community serosurvey in December 2020, overall seroprevalence of SARS-CoV-2 antibodies was 27.7%. The highest age-stratified seroprevalence was observed in participants aged 60-69 years (45.5%, 95% CI: 32.3% to 58.6%). The lowest was in children aged 0-9 years (14.0%, 95% CI: 7.4% to 20.6%).

## *Conclusions*

SARS-CoV-2 in rural Mali is much more widespread than assumed by national testing data and particularly in the older population and frontline HCWs. The observation is contrary to the widely expressed view, based on limited data, that COVID-19 infection rates were lower in 2020-2021 in West Africa than in other settings.




For peer review only

For peer review only - http://bmjopen.bmj.com/site/about/guidelines.xhtml

## Strengths and limitations of this study

- Our study was strengthened by the data available on participants from health facility clients, HCWs and the general population.
- We investigated both active infection and seroprevalence for HCWs and the general population during the same time period.
- First study of its kind in West Africa to provide routine surveillance data capturing.
- Our study was performed in one region and therefore it only reflects the COVID-19 situation of the Kita region.
- Extending active infection RT-PCR testing to non-symptomatic cases, both at the health facility and in the general population, would have provided more robust insights on infection rates as well as the level of asymptomatic cases.

For peer review only


For peer review only - http://bmjopen.bmj.com/site/about/guidelines.xhtml

# Introduction

The global coronavirus disease 2019 (COVID-19) pandemic, caused by the severe acute respiratory syndrome coronavirus 2 (SARS-CoV-2), has had a devastating impact on the health of populations in all regions of the world, leading to over five million deaths and tens of millions of cases, also causing widespread and long-term social and economic damage.[1] Despite predictions made during the so-called "first wave" (March-May 2020) that COVID-19 would spread widely and cause untold damage to communities in African countries, data during that period suggested that transmission, case, and death rates have been lower in African settings than in other regions and continents.[2,3]

Whereas the reported numbers of SARS-CoV-2 infection have been low in large parts of Africa, there is some evidence that this may be due to undertesting or underreporting. A recent Zambian post-mortem study suggested that many people who die have an undiagnosed SARS-CoV-2 infection.[4] Various studies have determined the seroprevalence in Africa.[5–13] A recent systematic review has estimated it to be approximately 22%, with highest seroprevalence in central Africa.[5] A recent study in Mali, conducted between November 2020 and June 2021, has reported a seroprevalence of 61.8% in health care workers (HCWs) who were in direct contact with COVID-patients in Bamako Hospital.[12] However, little is known about the spread of SARS-CoV-2 in rural areas among HCWs and the general population.

The objective of our study was to characterise the epidemiology of COVID-19 epidemic in Kita region in western Mali, an area with very low rates of reported infections. To this end, we monitored time-trends in the proportion of positive SARS-CoV-2 virus test results among patients presenting with suspected SARS-CoV-2 infection in primary health centres over a period of 10 months and complemented the data with seropositivity for SARS-CoV-2 antibodies among personnel working in the same facilities during the same time frame. Furthermore, in the middle



For peer review only - http://bmjopen.bmj.com/site/about/guidelines.xhtml

of the monitored time period, we conducted a population based viral and serological survey, to establish a point-prevalence of acute infection and past exposure to it, among apparently healthy people of different ages.

# Methods

## Study design and participants

The present study was designed to support the Malian Ministry of Health and Social Affairs' strategy to strengthen epidemiological surveillance and enhance the national response in Mali. The study was conducted in Kita region, Western Mali. The area is served by one referral health centre in Kita centre, while most health areas have a community health centre, offering a range of basic medical services with fee charged.

Our study targeted three groups –patients at a health facility, HCWs and apparently healthy community residents. We conducted surveillance for patients presenting at 1 referral health centre and 11 community health centres, over a period of 12 months. These patients presenting with symptoms of COVID-19 (respiratory symptoms and fever) were tested for active SARS-CoV-2 virus infection.

We conducted a HCWs serosurvey in five of the same health facilities over a period of 9 months and community serosurvey at one time point in 16 villages. Between September 2020 and May 2021, a total of 113 HCWs were approached in five health facilities and invited to participate in the HCWs SARS-CoV-2 serosurvey. The HCWs samples were from healthy nurses, doctors, office



For peer review only - http://bmjopen.bmj.com/site/about/guidelines.xhtml

workers, laboratory personnel and other auxiliary staff who consented to participate in the study. Among them, 78.8% were nurses reflecting their high proportion among HCWs.

The community samples were selected on the basis of cluster sampling. We used enumeration areas as clusters and determined the number of households from each cluster based on its recorded population size. The actual households to be invited were selected by random sampling. The sample size was originally calculated to estimate the seroprevalence of SARS-Cov-2 antibodies with an absolute precision (margin of error) of 2.5% and assuming a baseline prevalence of 5%. Accounting for clustering effects of households within villages and of individuals within households, we estimated to require a minimum sample size of 3000. Assuming an average village population of 1446 (based on available regional statistics), average household size of 14, and average number of under-5 children per household of 3, we decided to visit 215 households to enrol the study sample. In December 2020, a total of 229 randomly selected households were visited in villages in or near the Kita centre and invited to participate in a SARS-CoV-2 community survey. Heads of all the invited households provided a verbal consent and a total of 905 participants presented in the house during the visit were included in the survey.

## Data and Specimen collection

For the patient surveillance study, the study nurses filled in a patient screening form, a suspected infection enrolment form on exposures and symptoms and consenting form, followed by a medical examination and a flocked nasopharyngeal swab (NPS, flexible minitip, Copan) or oropharyngeal swab (OPS, flexible minitip, Copan; nasal and throat flocked swab, Dewei Medical Equipment Co., Ltd) specimen collection from those with a suspected SARS-CoV-2 infection, eg. patients experiencing fever, cough, shortness of breath and seeking medical advice. For the HCWs sero-surveillance study, study nurses filled in a screening, consent and enrolment form, a suspected



For peer review only - http://bmjopen.bmj.com/site/about/guidelines.xhtml

infection enrolment form on symptoms and exposure questionnaire. Four ml of venous blood sample (BD vacutainer, Cat. No. 369032) was collected monthly and an NPS or OPS was taken at enrolment and every 12 weeks thereafter. From each community survey participant, the nurse/data collector filled in a symptom and exposure questionnaire and collected a nasal swab specimen and a blood sample. A study nurse or a data collector filled out the symptom and exposure questionnaire using a REDCap electronic data capture application. The biological samples were collected by a doctor at the health centre and by a nurse in the community survey. The collected swab samples were transported at 2-8 °C in 3 ml PBS/VTM from Kita to Bamako lab for analysis. The blood samples were centrifuged, and serum was aliquoted into cryovials at the Kita lab and batched samples were transported in dry shipper to Bamako for analysis.

## Nucleic acid extraction and real time RT-PCR

RNA was extracted from sample swabs using a QIAamp Viral RNA Mini kit (Qiagen, Germany, Cat. No. 52906) according to the manufacturer's instructions.

Real time reverse transcription polymerase chain reaction (rtRT-PCR) assays for SARS-CoV-2 were carried out at the CVD-Mali Influenza laboratory, a participant in the WHO External Quality Assessment for the Detection of Influenza and SARS-CoV2 viruses. Briefly, extracted RNA was amplified using the Superscript One-step RT-PCR kit (Invitrogen, Thermo Fischer Scientific, Cat No. 11732-088) targeting the RdRp gene (Corman VM, 2020) or the Quantabio qScript XLT One-Step RT-qPCR ToughMix targeting the N-gene (N2 primers) (CDC assay, https://www.fda.gov/media/134922/download).

All assays were validated using WHO RNA samples received as part of an external Quality Assessment Programme and a panel of known positive and negative samples from University College London, UK.



For peer review only - http://bmjopen.bmj.com/site/about/guidelines.xhtml

## Serological assays

A laboratory technician performed the Wantai anti-SARS-Cov-2 total antibody ELISA (target: RBD, qualitative assay detecting IgM and IgG., Beijing Wantai Biological Pharmacy Ent, Beijing, China) following the manufacturer's instructions. The sensitivity and specificity reported was 93% and 100%, respectively. Serum samples (100 µL) from each participant were tested as a singlet. Ten batches were performed for community survey samples, six batches for HCW samples and one for repeated samples.

Assay validation was carried out using the WHO international standard (NIBSC code: 20/136), the WHO International Reference Panel for anti-SARS-CoV-2 immunoglobulin (NIBSC code: 20/268), and an anti-SARSCoV-2 Verification Panel for Serology Assays (NIBSC code: 20/B770). In addition, 100 pre-pandemic samples collected in 2012 were tested to assess the specificity of assay. All of these pre-pandemic serum samples were from adult women (mean age 26 years old, range 16-41). Following the manufacturer's instructions, samples giving an absorbance equal to or greater than the cut-off value (A/CO >=1) were considered positive. Samples with A/CO ratio between 0.9 and 1.1 were considered borderline and were retested.

## Statistical analysis

We compared proportions, means and standard deviations (SD) between groups by using Student's *t* test for continuous variables and Fisher's exact test for proportions and Chi-squared test for global difference with $p < 0.05$ indicating statistical significance. We calculated odds ratios (OR) and 95% confidence intervals (CI) with regression model. All statistical analyses were carried out using R versions 3.4.4 and 4.1.0.



For peer review only - http://bmjopen.bmj.com/site/about/guidelines.xhtml

## Patient and public involvement

Village chefs and health centre leaders were consulted before the study to ensure feasibility of the planned study procedures. Participants were not involved in the development of the research design, conduct of the research or preparation of the manuscript.

## Results

Between June 2020 and May 2021, a total of 13,104 people presenting at outpatient clinics or using preventive services at 12 health facilities in or near the town of Kita were screened for exposure or symptoms of SARS-CoV-2 infection. Of these, 2393 were deemed to have a suspected SARS-CoV-2 infection and 2392 agreed to provide nasopharyngeal or oropharyngeal samples for viral detection.

From all the tested samples from health centre patients, 68 (2.8%, 95% CI 2.2% - 3.6%) gave a positive test result for SARS-CoV-2 by RT-PCR. Prior to August 2020, no SARS-CoV-2 RNA could be detected, infection rates steadily rose from November 2020 onwards, peaking at 6.2% (95% CI 3.3% - 10.4%) in January 2021, and then gradually declined to 0.8% (95% CI 0.02% - 4.1%) in May 2021 (Figure 1). As shown in the figure, the increase in the proportion of positive samples followed a similar time trend in nationally reported numbers of SARS-CoV-2 infections and associated deaths.

Among the 113 invited staff members who all agreed to participate, 89 (78.8%) were nurses, 9 (7.9%) were doctors, 7 (6.2%) were administrative or laboratory technicians, and 8 (7.1%) were auxiliary staff including housekeepers, cleaners and security staff.



For peer review only - http://bmjopen.bmj.com/site/about/guidelines.xhtml

Over the 9-month follow-up period, the participants provided a total of 397 blood samples, with a range of 1-7 samples per participant. Of these samples, 175 (44.1%, 95% CI 39.1% - 49.1%) were positive for SARS-CoV-2 antibody by ELISA. By calendar month, the proportion of positive samples was approximately 10% until November 2020, increasing rapidly to 59.1% (95% CI 38.5% - 79.6%) in January 2021 and remained relatively constant thereafter (Figure 2). For different health professional groups, the proportion of SARS-CoV-2 antibody positive samples was highest among doctors (50.0%, 95% CI 29.9% - 70.1%, 13/26 samples), followed by 49.8% (95% CI 43.4% - 56.2%, 124/249) among nurses, 35.9% (95% CI 21.2% - 52.8%, 14/39) among administrative staff and laboratory technicians and 15.4% (95% CI 1.9% - 45.5%, 2/13) among auxiliary staff.

Of the 101 NPS or OPS samples collected from the health personnel, only one (1.0%) collected in November 2020 gave a positive test result for SARS-CoV-2 by RT-PCR.

Of the 905 community survey participants, 564 (62.3%) were women, 654 (72.3%) were at least 15 years old, 102 (11.3%) had respiratory symptoms in the preceding 14 days, 496 (55.4%) had attended a mass gathering in the preceding 3 months, none reported a contact with a COVID-19 patient, and 21 (2.3%) had a fever. 882 provided a blood sample for antibody analysis and 894 provided a nasal swab for viral detection. Among all the participants, the overall seroprevalence was 27.7% (95% CI 24.7% – 30.8%). The seroprevalence was lowest (14.0%, 95% CI: 7.4% - 20.6%) among 0-9-year-old and highest (45.5%, 95% CI: 32.3% to 58.6%) among 60-69-year-old participants (Figure 3). Participants who had attended a mass gathering in the preceding 3 months had a higher seroprevalence than those who had not (relative risk 1.48, 95% CI: 1.09 - 2.02). Participant sex, measured fever, and recent history of fever or other respiratory symptoms were not associated with the presence of SARS-CoV-2 antibodies in the blood samples (Table 1).



For peer review only - http://bmjopen.bmj.com/site/about/guidelines.xhtml

A total of 21 (2.4%, 95% CI 1.5% – 3.6%) community survey participants had a positive RT-PCR result for SARS-CoV-2. The proportion of positive SARS-CoV-2 PCR tests was lowest (0.4%, 95% CI: 0% - 1.3%) among 10-19-year-old and highest (5.8%, 95% CI: 0.3% - 11.3%) among 50-59-year-old participants (Figure 3).

Of the 100 banked serum samples collected before the COVID-19 pandemic (in year 2012), one gave a borderline positive result and 99 gave a negative test result in the Wantai SARS-CoV-2 antibody test.

For peer review only

## Discussion

This study measured active and past COVID-19 infection in Malian HCWs and the general population through RT-PCR and seroprevalence testing. The results point towards significant underestimation of Mali's COVID-19 infection rates, as measured by current testing mechanisms. The study also provides insights into COVID-19 transmission patterns and risk factors. Extremely high infection rates were demonstrated by the study's serological testing. By December 2020 over a quarter (27.7%) of Kita's general population, and more than a third (41.9%) of HCWs had been infected. By January 2021, this had increased to 60% of HCWs.

National routine surveillance morbidity data for the study period (June 2020 – May 2021), indicates that in June, transmission was at its first-wave peak, and that two later larger waves occurred, peaking in early December 2020 and April 2021.[14] Both the study's facility-based client/patient active infection investigation and HCWs seroprevalence findings indicate that Kita region's main transmission started later, in November 2020 and at the beginning it was very low, but increased gradually. Both study groups indicate a peak in December/January, similar to that seen nationally. The clients' active infection rate then showed a gradual decline to May rather



For peer review only - http://bmjopen.bmj.com/site/about/guidelines.xhtml

than a second wave in March that was seen nationally.[14] This could be explained by containment measures reducing the emergence of a second peak in the general population.

A comparison of this study's seroprevalence and active infection rates show that seroprevalence is much higher than active cases in both the study's general population and in HCWs. The fact that this high seroprevalence did not translate into a similar expression of morbidity and resultant death, neither in our study nor in the national surveillance system is an interesting area to investigate. This scenario has been documented quite widely in Africa.[15,16] This could be explained by a multitude of interlinking factors. High levels of asymptomatic cases, both for the general population and HCWs, have been documented in African as well as global studies.[11,17–21] As our study's active infection testing selected suspected cases of COVID-19 only, we could have missed a large proportion of asymptomatic cases. National surveillance would also have missed these cases as, firstly, they wouldn't present to a health centre and, secondly, even those that did would have been missed by diagnostic protocols.

Mali has gaps in demographic, disease morbidity, and death surveillance, including for COVID-19. A recent Zambian study showed that nearly 20% of people who had died had a positive SARS-CoV-2 PCR test in a postmortem NP swab sample. These people had not been tested before death, even though some of them had presented with COVID-19 symptoms.[4] A similar situation is likely present in Mali where diagnostic capacity is limited, at 7191 tests per 1 million people (compared to that of South Africa, 113 756)and where COVID-19 stigma exists.[22,23]

This study showed that 1.5 times more HCWs in Kita had been infected with SARS-CoV-2 by December 2020 compared to the general population. In fact, half of patient facing cadres such as doctors and nurses had been exposed to the virus and this was higher than laboratory, administrative (both 36%) and auxiliary staff (15.4%). Measures of increased risk of infection in



For peer review only - http://bmjopen.bmj.com/site/about/guidelines.xhtml

HCWs vs the general population have been demonstrated in many global studies, with adequate PPE provision and use being key to minimizing this risk.[15,17,21,24] In resource-constrained settings such as Kita, key tools for protecting HCWs, along with early disease detection, are often limited or absent in Africa.[25,26] Measures to improve protection for doctors and nurses, especially considering low levels of health staff availability in the country – in 2017 Mali had only six qualified providers per 10,000 inhabitants – would be pertinent.[27]

In the general population, risk factors included increased age and attendance at mass gatherings. Seroprevalence was lowest in the very young, and highest in 60-69-year-olds but again lower in the 70+ year olds. The possible reasons for lower seroprevalence in young children might be rapid waning of antibodies following mild symptoms and less exposures compared with adults. Mass gatherings are likely sources of infection given COVID-19's transmission dynamics and the difficulty of ensuring appropriate prevention control measures (mask wearing, social distancing, hand washing) at such events. Interestingly, neither fever (current or recent) nor other respiratory symptoms were seen to be significant risk factors, which again infers a high asymptomatic population.

The study's principal strength is that we included health facility clients, HCWs and the general population. We also investigated both active infection and seroprevalence for HCWs and the general population during the same time period. Study limitations include restriction to one region and therefore this study only reflects the COVID-19 situation of the Kita region. Extending active infection RT-PCR testing to non-symptomatic cases, both at the health facility and in the general population, would have provided more robust insights on infection rates as well as the level of asymptomatic cases. Widening the study to investigate exposure/transmission factors for study participants, such as PPE provision and use, social distancing, COVID-19 interventions, and



For peer review only - http://bmjopen.bmj.com/site/about/guidelines.xhtml

behaviour change would have allowed the study to explore risk factors to better inform policy. When interpreting the RT-PCR results, possible sources of bias could have occurred within the sample collection regarding sample types (oropharyngeal/nasopharyngeal swabs), sample quality, transportation and temperature, and also in the use of 3 different RT-PCR assays. The latter is unlikely to have affected the results as all RT-PCR assays were validated in-house and by others.[28] Previous reports have suggested that some SARS-CoV-2 ELISA kits lack specificity when used on serum samples originating from Africa and in particular exhibit pre-existing cross-reactivity to SARS-CoV-2.[29,30] Our assay showed a 99% specificity when pre-pandemic samples were analysed and previous work has shown the Wantai assay to be highly sensitive (93%) and specific (100%).[31]

In conclusion, SARS-CoV-2 in rural Mali is much more widespread than assumed by national testing data and particularly in the older population and frontline health workers. The observation is contrary to the widely expressed view, based on limited data, that COVID-19 infection rates were lower in 2020-2021 in West Africa than in other settings.

Institutional preparedness as well as public risk assessment and personal protection capacity should be augmented, and behaviours in high-risk activities such as mass gatherings improved, if cessation of such events is not possible. Improving the understanding of COVID-19 in Mali through surveillance will enable the country to reduce the economic and social impacts of the disease. Further studies which provide accurate estimates of SARS-CoV-2 infection rates and seroconversion in the population would enable the development of a national transmission model, mapping of transmission hotspots, identification of national/regional risk factors, and the prioritization of equitable and effective responses. Such studies could also direct policy determinations in terms of disease prioritization, ensuring that resources reallocated for COVID-19 control measures are used appropriately.



For peer review only - http://bmjopen.bmj.com/site/about/guidelines.xhtml

For peer review only

## Contributors

DA and FCH contributed equally and share first authorship. CD and YMF contributed equally and share last authorship. JL performed statistical analysis. PA, UA, NK, SS, KW, CD and YMF initiated the project and preliminary design. DA, FCH, LA, HB, ECG, FD, RI, OM, UM, OS, AT developed the design and methods. DA, FCH, CD and YMF wrote the first draft of the manuscript. All authors contributed to the design of the study and interpretation of its results, and revised and approved the manuscript. PA and YMF are the guarantors and accept full responsibility for the work and the conduct of the study, had access to the data, and controlled the decision to publish. The corresponding author attests that all listed authors meet authorship criteria and that no others meeting the criteria have been omitted.

## Data sharing

Deidentified participant data might be available on reasonable request to the corresponding author.

## Ethics

The study protocol was approved by the Faculté de Médecine, Pharmacie et d'Odonto-stomatologie (FMPOS) institutional review board in Mali and only participants who provided informed consent were enrolled in the study. All consents were requested verbally and recorded in the electronic data capture system. The minors' caregivers gave consent for their children to participate in the study.

## Funding

The study was funded by Bill & Melinda Gates Foundation, grant number INV-017141. The funders had no role in study design, data collection and analysis, decision to publish, or preparation of the manuscript.

## Competing interests

All authors have completed the ICMJE uniform disclosure form at www.icmje.org/coi_disclosure.pdf and declare: PA had financial support from Bill & Melinda Gates Foundation for the submitted work; no financial relationships with any organisations that might have an interest in the submitted work in the previous three years; no other relationships or activities that could appear to have influenced the submitted work.

For peer review only

## References


1 Weekly epidemiological update on COVID-19 - 7 December 2021. https://www.who.int/publications/m/item/weekly-epidemiological-update-on-covid-19---7-december-2021.

2 Africa CDC - COVID-19 Daily Updates. Africa CDC. https://africacdc.org/covid-19/.

3 WHO Coronavirus (COVID-19) Dashboard. https://covid19.who.int.

4 Mwananyanda L, Gill CJ, MacLeod W, *et al.* Covid-19 deaths in Africa: prospective systematic postmortem surveillance study. *BMJ* 2021;:n334. doi:10.1136/bmj.n334

5 Chisale MRO, Ramazanu S, Mwale SE, *et al.* Seroprevalence of anti-SARS-CoV-2 antibodies in Africa: A systematic review and meta-analysis. *Reviews in Medical Virology* 2022;**32**:e2271. doi:10.1002/rmv.2271

6 Okpala OV, Dim CC, Ugwu CI, *et al.* Population seroprevalence of SARS-CoV-2 antibodies in Anambra State, South-East, Nigeria. *International Journal of Infectious Diseases* 2021;**110**:171–8. doi:10.1016/j.ijid.2021.07.040

7 Barrie MB, Lakoh S, Kelly JD, *et al.* SARS-CoV-2 antibody prevalence in Sierra Leone, March 2021: a cross-sectional, nationally representative, age-stratified serosurvey. *BMJ Global Health* 2021;**6**:e007271. doi:10.1136/bmjgh-2021-007271

8 Fryatt A, Simms V, Bandason T, *et al.* Community SARS-CoV-2 seroprevalence before and after the second wave of SARS-CoV-2 infection in Harare, Zimbabwe. *eClinicalMedicine* 2021;**41**. doi:10.1016/j.eclinm.2021.101172

9 Uyoga S, Adetifa IMO, Otiende M, *et al.* Prevalence of SARS-CoV-2 Antibodies From a National Serosurveillance of Kenyan Blood Donors, January-March 2021. *JAMA* 2021;**326**:1436–8. doi:10.1001/jama.2021.15265

10 Sykes W, Mhlanga L, Swanevelder R, *et al.* Prevalence of anti-SARS-CoV-2 antibodies among blood donors in Northern Cape, KwaZulu-Natal, Eastern Cape, and Free State provinces of South Africa in January 2021. *Res Sq* 2021;:rs.3.rs-233375. doi:10.21203/rs.3.rs-233375/v1

11 Gelanew T, Seyoum B, Mulu A, *et al.* High Seroprevalence of Anti-SARS-CoV-2 Antibodies Among Ethiopian Healthcare Workers. *Res Sq* 2021;:rs.3.rs-676935. doi:10.21203/rs.3.rs-676935/v1

12 Somboro AM, Cissoko Y, Camara I, *et al.* High SARS-CoV-2 Seroprevalence among Healthcare Workers in Bamako, Mali. *Viruses* 2022;**14**:102. doi:10.3390/v14010102

13 El-Sokkary RH, Daef E, El-Korashi LA, *et al.* Sero-prevalence of anti-SARS-CoV-2 antibodies among healthcare workers: A multicenter study from Egypt. *Journal of Infection and Public Health* 2021;**14**:1474–80. doi:10.1016/j.jiph.2021.09.011

14 Mali: WHO Coronavirus Disease (COVID-19) Dashboard With Vaccination Data. https://covid19.who.int (accessed 10 Nov 2021).




For peer review only - http://bmjopen.bmj.com/site/about/guidelines.xhtml

15 Chibwana MG, Jere KC, Kamn'gona R, *et al.* High SARS-CoV-2 seroprevalence in health care workers but relatively low numbers of deaths in urban Malawi. *medRxiv* 2020;:2020.07.30.20164970. doi:10.1101/2020.07.30.20164970

16 Uyoga S, Adetifa IMO, Karanja HK, *et al.* Seroprevalence of anti–SARS-CoV-2 IgG antibodies in Kenyan blood donors. *Science* 2021;**371**:79–82. doi:10.1126/science.abe1916

17 Abatan B, Agboghoroma O, Akemoke F, *et al.* Intense and Mild First Epidemic Wave of Coronavirus Disease, The Gambia. *Emerg Infect Dis* 2021;**27**:2064–72. doi:10.3201/eid2708.204954

18 Paleker M, Tembo YA, Davies M-A, *et al.* Asymptomatic COVID-19 in South Africa – implications for the control of transmission. *Public Health Action* 2021;**11**:58–60. doi:10.5588/pha.20.0069

19 Fai KN, Corine TM, Bebell LM, *et al.* Serologic response to SARS-CoV-2 in an African population. *Scientific African* 2021;**12**:e00802. doi:10.1016/j.sciaf.2021.e00802

20 Oran DP, Topol EJ. Prevalence of Asymptomatic SARS-CoV-2 Infection. *Ann Intern Med* 2020;**173**:362–7. doi:10.7326/M20-3012

21 Olayanju O, Bamidele O, Edem F, *et al.* SARS-CoV-2 Seropositivity in Asymptomatic Frontline Health Workers in Ibadan, Nigeria. *The American Journal of Tropical Medicine and Hygiene* 2021;**104**:91–4. doi:10.4269/ajtmh.20-1235

22 Salyer SJ, Maeda J, Sembuche S, *et al.* The first and second waves of the COVID-19 pandemic in Africa: a cross-sectional study. *The Lancet* 2021;**397**:1265–75. doi:10.1016/S0140-6736(21)00632-2

23 Seidu A-A, Hagan JE, Ameyaw EK, *et al.* The role of testing in the fight against COVID-19: Current happenings in Africa and the way forward. *International Journal of Infectious Diseases* 2020;**98**:237–40. doi:10.1016/j.ijid.2020.06.089

24 Pollán M, Pérez-Gómez B, Pastor-Barriuso R, *et al.* Prevalence of SARS-CoV-2 in Spain (ENE-COVID): a nationwide, population-based seroepidemiological study. *The Lancet* 2020;**396**:535–44. doi:10.1016/S0140-6736(20)31483-5

25 Galanis P, Vraka I, Fragkou D, *et al.* Seroprevalence of SARS-CoV-2 antibodies and associated factors in healthcare workers: a systematic review and meta-analysis. *J Hosp Infect* 2021;**108**:120–34. doi:10.1016/j.jhin.2020.11.008

26 Quadri NS, Sultan A, Ali SI, *et al.* COVID-19 in Africa: Survey Analysis of Impact on Health-Care Workers. *Am J Trop Med Hyg* 2021;**104**:2169–75. doi:10.4269/ajtmh.20-1478

27 M'Bo M, Tessougue J, International I, *et al.* DECENTRALIZING MALI'S HEALTH WORKFORCE INFORMATION SYSTEM. ;:4.

28 Brown JR, O'Sullivan DM, Shah D, *et al.* Comparison of SARS-CoV-2 N gene real-time RT-PCR targets and commercially available mastermixes. *J Virol Methods* 2021;**295**:114215. doi:10.1016/j.jviromet.2021.114215



For peer review only - http://bmjopen.bmj.com/site/about/guidelines.xhtml

29 Emmerich P, Murawski C, Ehmen C, *et al.* Limited specificity of commercially available SARS-CoV-2 IgG ELISAs in serum samples of African origin. *Trop Med Int Health* 2021;:10.1111/tmi.13569. doi:10.1111/tmi.13569

30 Tso FY, Lidenge SJ, Peña PB, *et al.* High prevalence of pre-existing serological cross-reactivity against severe acute respiratory syndrome coronavirus-2 (SARS-CoV-2) in sub-Saharan Africa. *Int J Infect Dis* 2021;**102**:577–83. doi:10.1016/j.ijid.2020.10.104

31 Lassaunière R, Frische A, Harboe ZB, *et al.* Evaluation of nine commercial SARS-CoV-2 immunoassays. 2020. doi:10.1101/2020.04.09.20056325

For peer review only



For peer review only - http://bmjopen.bmj.com/site/about/guidelines.xhtml

**Table 1. Seroprevalence of SARS-CoV-2-specific total antibody by background characteristics**

| | Sample size, n | Seropositive participants, n | Seroprevalence, % | OR (95% CI) |
|---|---|---|---|---|
| Participants, *n* | 875 | 242 | 27.7% | |
| Sex | | | | |
| Male | 330 | 98 | 29.7% | Ref |
| Female | 543 | 142 | 26.2% | 0.84 (0.62-1.14) |
| Fever during past 14 days | | | | |
| No fever | 832 | 229 | 27.5% | Ref |
| Fever | 41 | 11 | 26.8% | 0.97 (0.48-1.96) |
| Fever (body temperature over 38 °C) | | | | |
| No fever | 852 | 238 | 27.9% | Ref |
| Fever | 21 | 2 | 9.5% | 0.27 (0.06-1.17) |
| Respiratory symptoms in the past 14 days | | | | |
| No symptoms | 773 | 216 | 27.9% | Ref |
| Any symptoms | 100 | 24 | 24.0% | 0.81 (0.50-1.32) |
| Mass gatherings in the past 3 months | | | | |
| No attendance | 370 | 86 | 23.0% | Ref |
| Attendance | 487 | 151 | 31.0% | 1.48 (1.09-2.02) |



For peer review only - http://bmjopen.bmj.com/site/about/guidelines.xhtml

**Legends of Figures:**

**Figure 1.** Percentage of SARS-CoV-2 positive RT-PCR test by month in routine surveillance patients from health facilities. Grey area and values in parentheses indicate 95% confidence interval from the proportion of SARS-CoV-2 RNA containing samples. Yellow lines indicate nationally reported numbers of SARS-CoV-2 infections ((WHO https://covid19.who.int/region/afro/country/ml). Values above the x-axis indicate number of study samples in each month. PS = pharyngeal swab, SARS-CoV-2 = Severe acute respiratory syndrome coronavirus-2, RNA = ribonucleic acid.

**Figure 2.** Seroprevalence of SARS-CoV-2-specific total antibody by month in health care worker serosurvey. Grey area and values in parentheses indicate 95% confidence interval from the proportion of SARS-CoV-2-specific antibody containing samples. Values above the x-axis indicate number of study samples in each month. SARS-CoV-2 = Severe acute respiratory syndrome coronavirus-2.

**Figure 3.** Seroprevalence of SARS-CoV-2-specific total antibody and percentage of SARS-CoV-2 positive RT-PCR test by 10-year age interval group in community participants. Red bars indicate proportion (95% CI) of serum samples containing SARS-CoV-2-specific antibodies (primary y-axis), Chi-squared global age group difference, p=0.002; blue bars indicate proportion (95% CI) of PS samples containing SARS-CoV-2 RNA (secondary y axis). PS = pharyngeal swab, SARS-CoV-2 = Severe acute respiratory syndrome coronavirus-2, RNA = ribonucleic acid.



For peer review only - http://bmjopen.bmj.com/site/about/guidelines.xhtml

**Figure 1.**

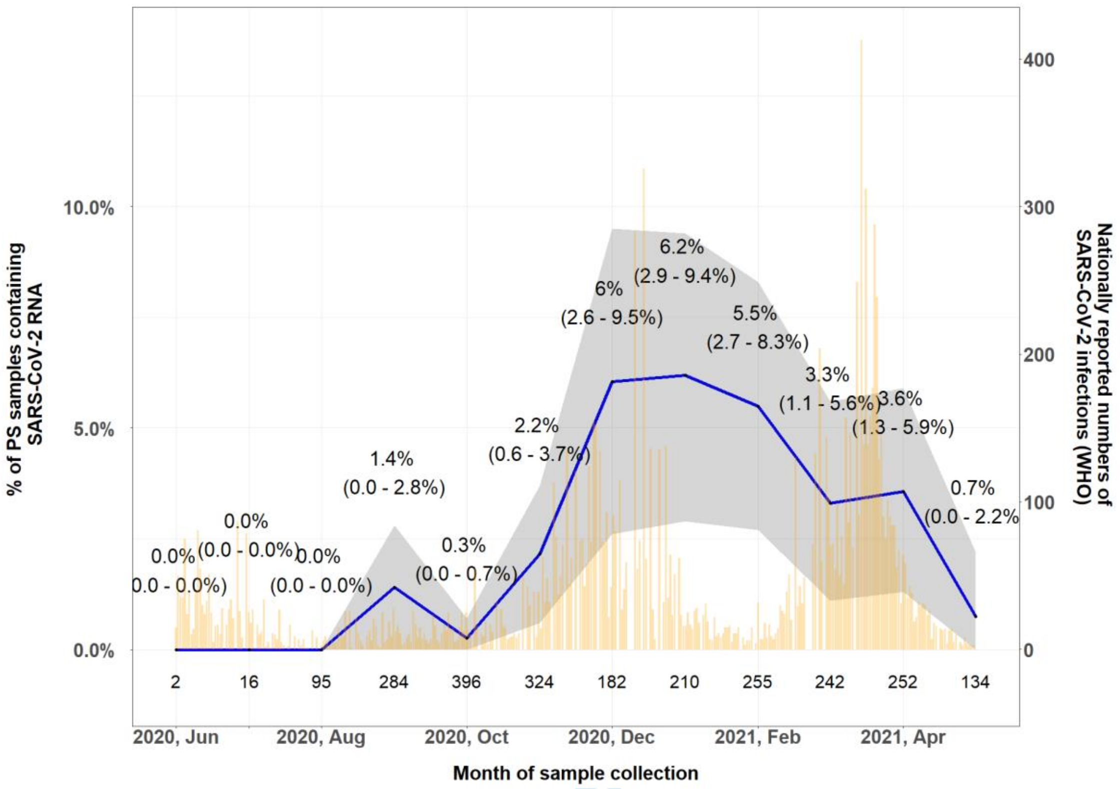


eview only

**Figure 2.**

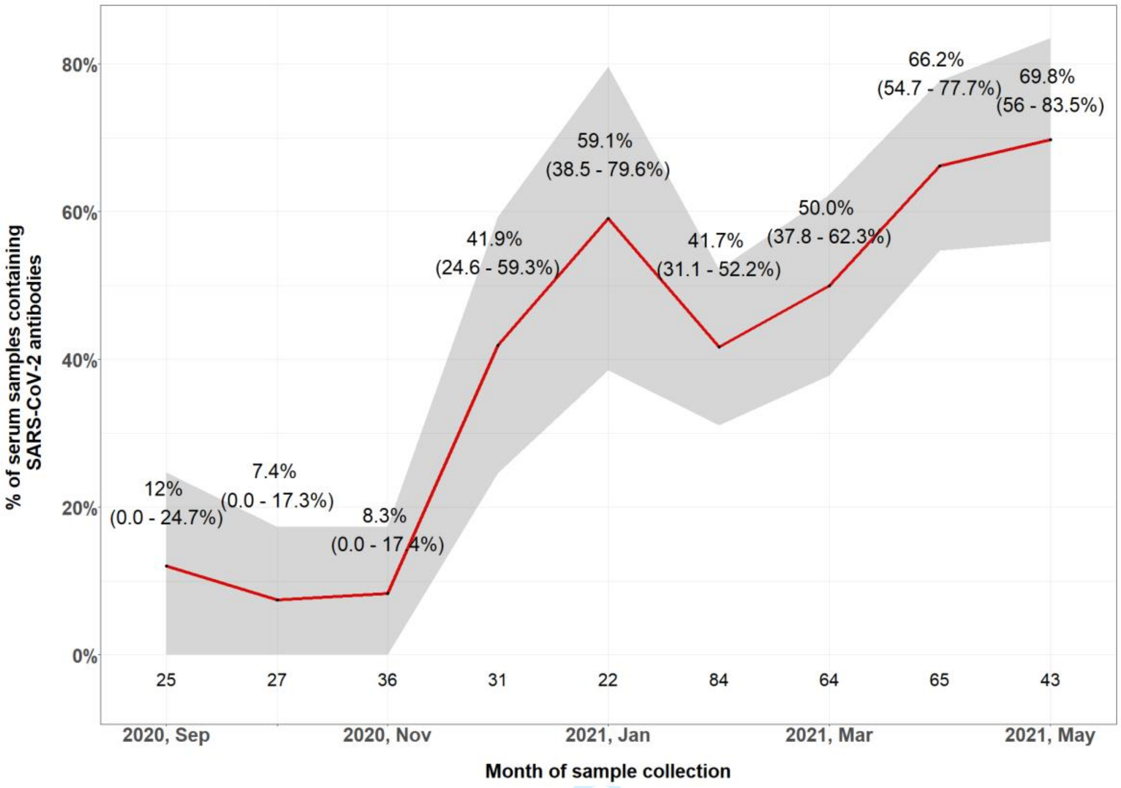
% of serum samples containing SARS-CoV-2 antibodies
80%
60%
40%
20%
0%
12% (0.0 - 24.7%)
7.4% (0.0 - 17.3%)
8.3% (0.0 - 17.4%)
41.9% (24.6 - 59.3%)
59.1% (38.5 - 79.6%)
41.7% (31.1 - 52.2%)
50.0% (37.8 - 62.3%)
66.2% (54.7 - 77.7%)
69.8% (56 - 83.5%)
25
27
36
31
22
84
64
65
43
2020, Sep
2020, Nov
2021, Jan
2021, Mar
2021, May
Month of sample collection

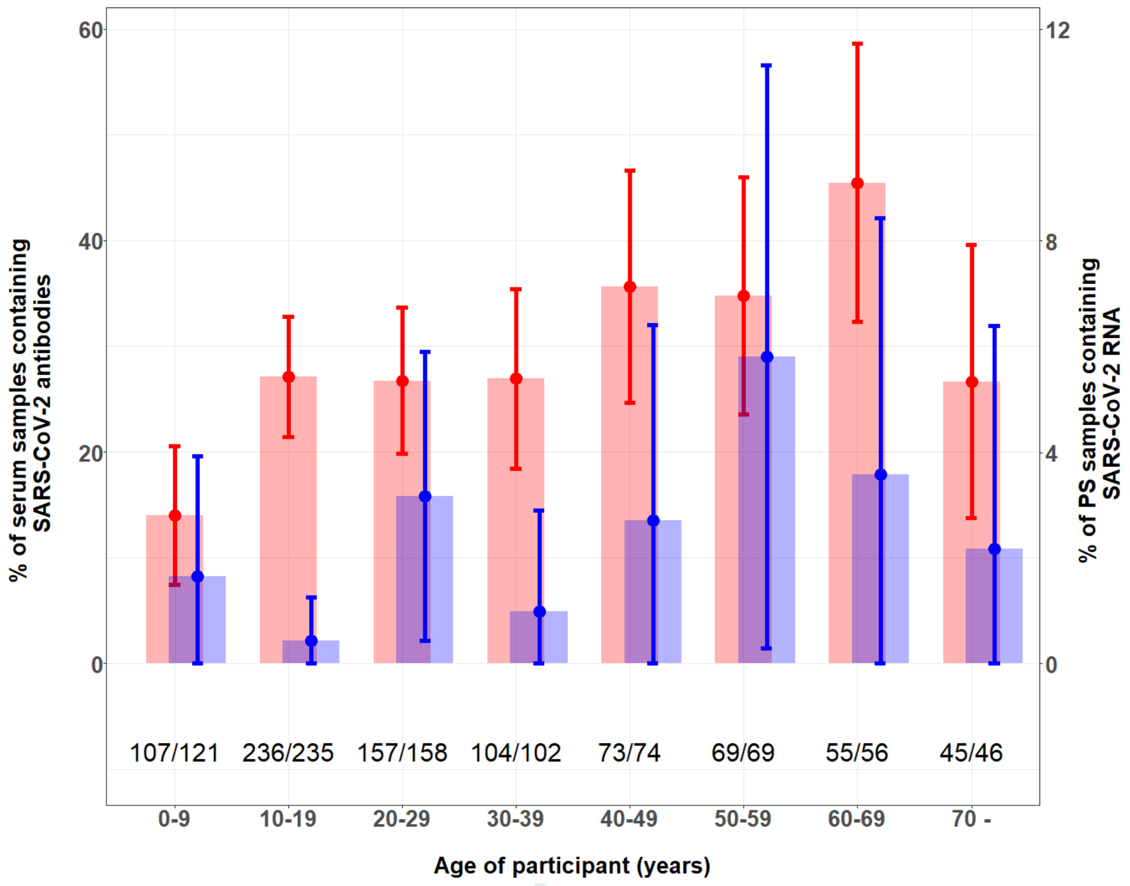
% of serum samples containing SARS-CoV-2 antibodies
% of PS samples containing SARS-CoV-2 RNA
60
40
20
0
12
8
4
0
107/121
236/235
157/158
104/102
73/74
69/69
55/56
45/46
0-9
10-19
20-29
30-39
40-49
50-59
60-69
70 -
Age of participant (years)

STROBE Statement—checklist of items that should be included in reports of observational studies

| | Item No | Recommendation | Page No |
|---|---|---|---|
| **Title and abstract** | 1 | (*a*) Indicate the study's design with a commonly used term in the title or the abstract | 1 |
| | | (*b*) Provide in the abstract an informative and balanced summary of what was done and what was found | 2 |
| **Introduction** | | | |
| Background/rationale | 2 | Explain the scientific background and rationale for the investigation being reported | 4 |
| Objectives | 3 | State specific objectives, including any prespecified hypotheses | 4 |
| **Methods** | | | |
| Study design | 4 | Present key elements of study design early in the paper | 5 |
| Setting | 5 | Describe the setting, locations, and relevant dates, including periods of recruitment, exposure, follow-up, and data collection | 5-6 |
| Participants | 6 | (*a*) *Cohort study*—Give the eligibility criteria, and the sources and methods of selection of participants. Describe methods of follow-up<br>*Case-control study*—Give the eligibility criteria, and the sources and methods of case ascertainment and control selection. Give the rationale for the choice of cases and controls<br>*Cross-sectional study*—Give the eligibility criteria, and the sources and methods of selection of participants | 5-6 |
| | | (*b*) *Cohort study*—For matched studies, give matching criteria and number of exposed and unexposed<br>*Case-control study*—For matched studies, give matching criteria and the number of controls per case | |
| Variables | 7 | Clearly define all outcomes, exposures, predictors, potential confounders, and effect modifiers. Give diagnostic criteria, if applicable | 5-8 |
| Data sources/ measurement | 8* | For each variable of interest, give sources of data and details of methods of assessment (measurement). Describe comparability of assessment methods if there is more than one group | *5-8* |
| Bias | 9 | Describe any efforts to address potential sources of bias | 5-8 |
| Study size | 10 | Explain how the study size was arrived at | 5-6 |
| Quantitative variables | 11 | Explain how quantitative variables were handled in the analyses. If applicable, describe which groupings were chosen and why | 8 |
| Statistical methods | 12 | (*a*) Describe all statistical methods, including those used to control for confounding | 8 |
| | | (*b*) Describe any methods used to examine subgroups and interactions | |
| | | (*c*) Explain how missing data were addressed | |
| | | (*d*) *Cohort study*—If applicable, explain how loss to follow-up was addressed<br>*Case-control study*—If applicable, explain how matching of cases and controls was addressed<br>*Cross-sectional study*—If applicable, describe analytical methods taking account of sampling strategy | |
| | | (*e*) Describe any sensitivity analyses | |

Continued on next page



For peer review only - http://bmjopen.bmj.com/site/about/guidelines.xhtml

For peer review only

| | | | |
|---|---|---|---|
| **Results** | | | |
| Participants | 13* | (a) Report numbers of individuals at each stage of study—eg numbers potentially eligible, examined for eligibility, confirmed eligible, included in the study, completing follow-up, and analysed | 9-10 |
| | | (b) Give reasons for non-participation at each stage | |
| | | (c) Consider use of a flow diagram | |
| Descriptive data | 14* | (a) Give characteristics of study participants (eg demographic, clinical, social) and information on exposures and potential confounders | 9-10 |
| | | (b) Indicate number of participants with missing data for each variable of interest | |
| | | (c) *Cohort study*—Summarise follow-up time (eg, average and total amount) | |
| Outcome data | 15* | *Cohort study*—Report numbers of outcome events or summary measures over time | *9-10* |
| | | *Case-control study*—Report numbers in each exposure category, or summary measures of exposure | |
| | | *Cross-sectional study*—Report numbers of outcome events or summary measures | |
| Main results | 16 | (*a*) Give unadjusted estimates and, if applicable, confounder-adjusted estimates and their precision (eg, 95% confidence interval). Make clear which confounders were adjusted for and why they were included | |
| | | (*b*) Report category boundaries when continuous variables were categorized | |
| | | (*c*) If relevant, consider translating estimates of relative risk into absolute risk for a meaningful time period | |
| Other analyses | 17 | Report other analyses done—eg analyses of subgroups and interactions, and sensitivity analyses | |
| **Discussion** | | | |
| Key results | 18 | Summarise key results with reference to study objectives | 11 |
| Limitations | 19 | Discuss limitations of the study, taking into account sources of potential bias or imprecision. Discuss both direction and magnitude of any potential bias | 13-14 |
| Interpretation | 20 | Give a cautious overall interpretation of results considering objectives, limitations, multiplicity of analyses, results from similar studies, and other relevant evidence | 11-13 |
| Generalisability | 21 | Discuss the generalisability (external validity) of the study results | 14 |
| **Other information** | | | |
| Funding | 22 | Give the source of funding and the role of the funders for the present study and, if applicable, for the original study on which the present article is based | 16 |

*Give information separately for cases and controls in case-control studies and, if applicable, for exposed and unexposed groups in cohort and cross-sectional studies.

**Note:** An Explanation and Elaboration article discusses each checklist item and gives methodological background and published examples of transparent reporting. The STROBE checklist is best used in conjunction with this article (freely available on the Web sites of PLoS Medicine at http://www.plosmedicine.org/, Annals of Internal Medicine at http://www.annals.org/, and Epidemiology at http://www.epidem.com/). Information on the STROBE Initiative is available at www.strobe-statement.org.

For peer review only
